# Adenosine Triggers an ADK-Dependent Intracellular Signaling Pathway Interacts PFKFB3-Mediated Glycolytic Metabolism to Promote Newly Formed Myofibers Development

**DOI:** 10.3390/ijms262412184

**Published:** 2025-12-18

**Authors:** Xiao Wu, Dawei Zeng, Baojia Wang, Jie Liu, Yue Zhang, Cong Huang, Qian Nie, Liangqin Shi, Yong Wang

**Affiliations:** 1 College of Basic Medicine, Chengdu University of Traditional Chinese Medicine, Chengdu 611137, China; 2 Hospital of Chengdu University of Traditional Chinese Medicine, School of Clinical Medicine, Chengdu University of Traditional Chinese Medicine, Chengdu 610075, China

**Keywords:** adenosine, ADK-dependent intracellular signaling pathway, PFKFB3-mediated glycolytic metabolism, newly myofiber development, skeletal muscle regeneration

## Abstract

Myopathy encompasses a group of diseases characterized by abnormalities in both muscle function and structure. However, the underlying regulatory mechanisms of newly formed myofiber development remain poorly defined. No promising therapeutic approach has been developed, but numerous medication options are available to alleviate symptoms. Our previous studies demonstrated that adenosine kinase (ADK) is critical in regulating adenosine metabolism, pathological angiogenesis, pathological vascular remodeling, and vascular inflammatory diseases. Adenosine dynamically distributes between extracellular and intracellular, and adenosine concentration regulates ADK expression. However, the mechanism by which adenosine triggers an ADK-dependent intracellular signaling pathway to regulate skeletal muscle regeneration is not well defined. This study aimed to evaluate whether the adenosine-induced intracellular signaling pathway is involved in regulating myopathy, and how it regulates the development of newly formed myofibers. In this study, an intramuscular injection of cardiotoxin was used to induce a skeletal muscle injury model; satellite cells and C2C12 cells were employed. Whether adenosine regulates satellite cell activity, new myofiber formation and differentiation, as well as fusion of myofibers, were determined by H&E staining, BrdU incorporation assay, and spheroid sprouting assay. Interaction between ADK and PFKFB3 was evaluated by IF staining, PPI network analysis, molecular docking simulation, and CO-immunoprecipitation assay. The results demonstrated that adenosine dynamically distributes between extracellular and intracellular through concentrative nucleoside transports or equilibrative nucleoside transporters, and it rapidly induces an ADK-dependent intracellular signaling pathway, which interacts with PFKFB3-mediated glycolytic metabolism to promote satellite cell activity, new myofiber formation, differentiation, and fusion, and eventually enhances skeletal muscle regeneration after injury stress. The remarkable endogenous regeneration capacity of skeletal muscle, which is regulated by adenosine-triggered intracellular signaling, presents a promising therapeutic strategy for treating muscle trauma and muscular dystrophies.

## 1. Introduction

Myopathy encompasses a group of diseases characterized by abnormalities in both muscle function and structure. Classical myopathy can be classified into two categories: inherited myopathy (primary myopathy) and acquired myopathy (secondary myopathy). Inherited myopathy includes polymyositis, dermatomyositis, muscular dystrophy, myasthenia gravis, amyotrophic lateral sclerosis, rhabdomyolysis, cardiomyopathy, sarcopenia, etc. Multiple causes, such as endocrine disorder [1], inflammatory response, infection diseases, metabolic disorder [2], immunological condition [3], hormonal impropriation [4], electrolyte imbalance, neurological disfunction, as well as abnormal genetic background, contribute to the development of myopathy [5]. Myopathy is characterized by structural and function impairment in skeletal muscle fibers. However, the underlying mechanism of the development of myopathy is not well defined. There is no therapeutic approach for myopathy, but many medication options are available to manage symptoms.

Adult skeletal muscles are composed of multinucleated myofibers, with myonuclear located at the periphery of myofibers. These myofibers are terminal differentiated and cannot re-enter cell cycle to replenish cell population after skeletal damage. However, adult skeletal muscle exhibits a remarkable capacity for regeneration following injury or disease. Following trauma or acute injury-induced damage, skeletal muscle exhibits degeneration, inflammatory response, and skeletal muscle regeneration phase [6,7]. Different cell types were involved in skeletal muscle regeneration, which included satellite cells, inflammatory cells, fibroblast cells, adipocyte, endothelial cells, and smooth muscle cells. Satellite cell is the extensively studied cell type and plays a pivotal role during skeletal muscle regeneration [8,9,10,11]. Satellite cells are quiescent cells, which are located between sarcolemma and basal lamina [12,13]. Injury or disease in skeletal muscle results in the activation of satellite cells, enhancing migration, promoting proliferation, and facilitating differentiation [7,14]. The newly formed myofibers can fuse with one another or with a surviving segment of the damaged skeletal myofibers, eventually regulating skeletal muscle regeneration that characterizes centrally located multiple nuclei. If not perfectly aligned between surviving myofibers and newly formed myofibers during skeletal muscle regeneration, it can progressively develop into chronic myopathy.

Skeletal muscle regeneration is a highly orchestrated biological process, which is essentially a satellite cell-activated response to injury stress. They express fibroblast growth factor receptor 2 to drive cell proliferation, which causes rapid onset expression of myogenic transcription factors MYF5 and MYOD1. The expression of MYOD1 in quiescent satellite cells cannot be detected [15]. However, the high expression level of MYOD1 was exhibited in activated satellite cells, which is maintained during the proliferation phase and early differentiation phase. Myogenin (MYOG) is not expressed either in quiescent satellite cells or proliferating undifferentiated myoblast, whereas its expression is dramatically increased in differentiated myoblast [16]. High expression levels of myogenic regulatory factors (MRFs) were exhibited in adult mature skeletal muscle. Some other myogenesis-related marker genes have been identified during skeletal muscle regeneration, including Myozenin (Myoz1 and Myoz3) [17], Myomaker [18], troponin1, and dystrophin. A high expression level is observed in skeletal muscle tissues, whereas it is not detectable in cultures’ myotubes.

Evidence indicated that VEGF enhances the growth of myogenic fibers and inhibits apoptosis of myogenic cells [19]. Some other causes are also critical in regulating skeletal muscle regeneration. Locally produced growth factors are critical in regulating satellite cell proliferation, differentiation, and fusion through anticrime or paracrine manner during myogenesis, including FGFs, PGDGs, IGFs and macrophage-derived growth factor (MDGF) [20,21]. Hormones, such as adrenocorticotropin, interferon, and dexamethasone, can also enhance satellite cell proliferation [22].

Adenosine, an endogenous purine nucleoside, can regulate multiple physiological processes through four known G-protein-coupled receptors, ADORA1, ADORA2A, ADORA2B, and ADORA3. Injury promotes expression of adenosine receptors, ADORA2B and ADORA3, which can attenuate ischemia and reperfusion-induced skeletal muscle damage [23,24,25,26]. ADORA2A suppresses apoptosis during myogenesis through inducing Bcl-2 expression [27]. Adenosine triphosphate reported to enhance post-ischemic survival of skeletal muscle [28]. Adenosine can be produced in both intracellular and extracellular, and it can be transported across members through concentrative nucleoside transports (SLC28A1, SLC28A2, SLC28A3) or equilibrative nucleoside transporters (SLC29A1, SLC29A2, SLC29A3, SLC29A4) [29]. Intracellular adenosine has a short half-life, which arises from catalytic reactions of multiple enzymes, including SAH-Hydrolase, adenosine Kinase, 5’-Nucleotidase, and adenosine deaminase [30]. Adenosine-associated intracellular signaling pathways play a critical role in regulating AMP-mediated bioenergy supplement [31], as well as in pathological angiogenesis, pathological vascular remodeling, and vascular inflammatory diseases [32,33,34]. Suppression of ADK-attenuated abdominal aortic aneurysm through Histone methylation of VSMC inflammation [35]. However, whether adenosine-associated intracellular signaling pathways can regulate skeletal muscle diseases is largely unknown.

Disorder of skeletal muscle glycogenolysis and glycolysis are responsible for many skeletal muscle diseases [36]. AMPK can regulate various aspects of mitochondrial biology and homeostasis [37]. AMP-activated protein kinase stimulates Warburg-like glycolysis and activation of satellite cells during skeletal muscle regeneration [38]. YY1 gene regulates satellite cell metabolic reprogramming through glycolytic pathway [39].

Intracellular adenosine can be generated by dephosphorylation of adenosine triphosphate or hydrolysis of S-adenosylhomocysteine. Adenosine can be converted to AMP via catalysis by ADK. Great achievements have been made in the research on adenosine metabolic pathways which associated pathological angiogenesis [32], vascular inflammation [35], neointima formation [34], and various nervous system diseases over the past few decades. This study aimed to evaluate whether adenosine induces intracellular signaling pathways involved in myopathy regulation and how it regulates the development of newly formed myofibers. The remarkable endogenous regenerative potential of skeletal muscle, which is regulated by adenosine associated intracellular signaling pathways, may represent a promising therapeutic strategy for muscle trauma and muscular dystrophies.

## 2. Results

### 2.1. Adenosine Induces Satellite Cell Activation and Promotes Satellite Cell Proliferation and Migration

**Figure 1:**
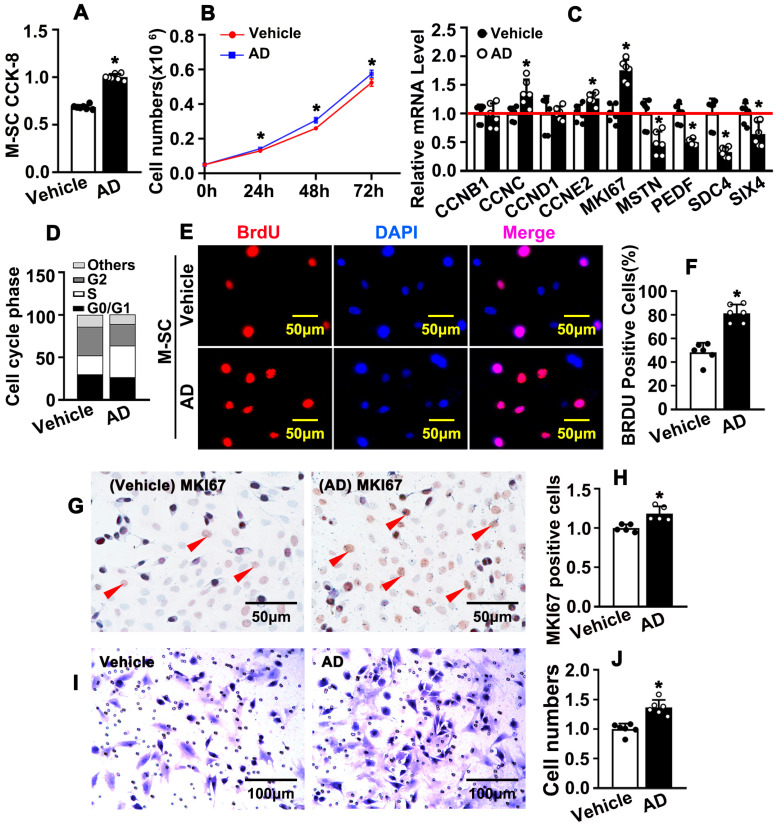
**Adenosine induces satellite cell activation and promotes satellite cell proliferation and migration.** (**A**) M-SC was treated with adenosine (10 μM) for 24 h (h), and viability was assessed by CCK-8 cell proliferation assay (*n* = 7; unpaired *t*-test). (**B**) M-SC numbers were counted after adenosine (AD) treatment for 24 h, 48 h, and 72 h (*n* = 6; unpaired *t*-test). (**C**) After 30 h of adenosine treatment, real-time PCR was performed to detect the expression level of mRNA in M-SC for cell cycle-associated genes (*n* = 6; unpaired *t*-test). (**D**) M-SC was treated with adenosine for 30 h and the cell cycle was determined by flow cytometry analysis after propidium iodide (PI) staining (*n* = 3; 2 × 10 ^4^ cells; unpaired *t*-test). (**E**) M-SC was treated with adenosine for 12 h, followed by incubation with BrdU reagent for 12 h, and IF staining was performed to detect BrdU incorporation. (**F**) Quantification of BrdU positive cells from (**E**) (*n* = 6; unpaired *t*-test). (**G**) After 30 h of adenosine treatment, proliferating M-SC was determined by IHC staining against MKI67 antibody (arrow: MKI67positive), and quantification of MKI67 positive cells exhibited (**H**) (*n* = 6; unpaired *t*-test). (**I**) Boyden chamber cell migration assay was performed to determine the migration of M-SC after adenosine treatment (*n* = 6; unpaired *t*-test), and migrated cells were quantified in (**J**). Data were presented as mean ± SEM, * *p* < 0.05 was considered significant.

Satellite cells are essential for skeletal muscle regeneration in responding to damage or myopathy. Quiescent satellite cells are rapid activated, recruited to injury area, proliferated, fused, and newly formed myofibers. PAX7 is a critical marker for activated satellite cells. To determine whether adenosine can promote satellite cell activity, we isolated murine satellite cell using enzyme-digested method. The purity of isolated satellite cells was confirmed by IF staining against PAX7 antibody (Supplementary Materials Figure S1A). Satellite cell viability and cell number were conspicuously increased after Adenosine treatment, which were determined by CCK-8 assay and cell number account experiment (Figure 1A,B and Supplementary Materials Figure S1B). Real-time PCR performed to evaluate cell cycle related genes, and transcription levels of CCNC, CCNE2, and MKI67 significantly increased, whereas MSTN, PEDF, SDC4, SIX4, CDKN2B, CDKN2A, CHKN2C, and CDKN1B dramatically decreased after adenosine treatment (Figure 1C and Supplementary Materials Figure S1C). Adenosine treatment induced satellite cells to enter synthesis phage during cell cycle, which was determined by flow cytometry after PI staining (Figure 1D and Supplementary Materials Figure S1D). We performed BrdU incorporation assay and found that adenosine treatment significantly improved BrdU positive cells (Figure 1E,F). We also performed IHC staining against MKI67 antibody and observed much more MKi67 positive cell exhibited in satellite cells after adenosine treatment (Figure 1G,H). We performed boyden chamber migration assay and our data indicated that adenosine enhanced satellite cell migration (Figure 1I,J). Our data indicated that adenosine induces satellite cell activation, as well as promote satellite cell proliferation and migration.

### 2.2. Adenosine Promotes C2C12 Cell Proliferation and Migration

**Figure 2:**
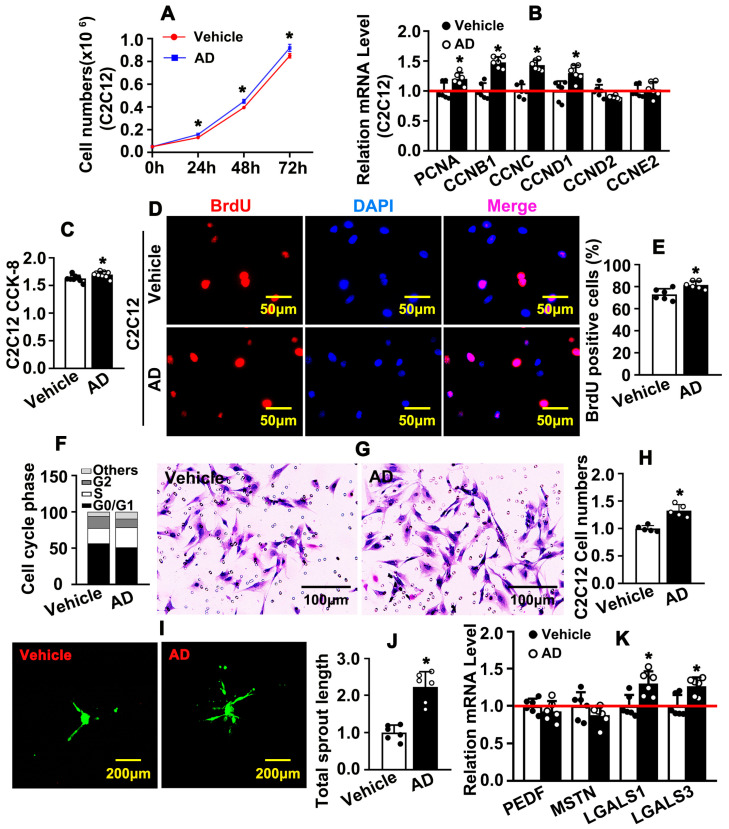
**Adenosine promotes C2C12 cell proliferation and migration.** (**A**) C2C12 cell number was counted after treatment with adenosine (10 μM) for 24, 48, and 72 h (*n* = 6; unpaired *t*-test). (**B**) C2C12 cells were treated with adenosine for 12 h and real-time PCR was performed to determine transcription levels of cell cycle related genes (*n* = 6; unpaired *t*-test). (**C**) Treatment of C2C12 cells with adenosine; viability was determined by CCK8 assay. (**D**) C2C12 cells were treated with adenosine for 12 h, followed by labeling with BrdU reagent for 6 h, and IF staining was performed to determine BrdU-incorporated cells and BrdU-positive cells exhibited in (**E**) (*n* = 6, unpaired *t*-test). (**F**) After 24 h of adenosine treatment, cell cycle was detected by cell cytometry analysis after PI staining (*n* = 3; 2 × 10 ^4^ cells; unpaired *t*-test). (**G**) After 12 h of adenosine treatment, migration of C2C12 was detected by Boyden chamber assay (*n* = 5), and migrated cells were quantified in (**H**) (*n* = 6; unpaired *t*-test). (**I**) The migratory ability of C2C12 after adenosine treatment was evaluated by spheroid assay, and sprouting lengths were quantified in (**J**) (*n* = 6; unpaired *t*-test). (**K**) Real-time PCR was performed to detect transcript levels of migration-related genes after adenosine treatment (*n* = 6; unpaired *t*-test). Data were presented as mean ± SEM, * *p* < 0.05 was considered significant.

C2C12 is an immortalized murine myoblast cell line. C2C12 can be rapidly proliferated under high-serum conditions and differentiated into myotubes under lower serum conditions. Whether adenosine can regulate C2C12 proliferation under high-serum conditions is not well defined. We first performed cell number counting and observed that adenosine can induce cell growth (Figure 2A). Our real-time PCR results indicated that adenosine can increase PCNA, CCNB1, CCNC, and CCND1 expression, while decreasing CDKN2A, CDKN2C, CDKN1C, and CDKN1B (Figure 2B and Supplementary Materials Figure S2A). We performed CCK8 assay and observed that adenosine can induce cell viability (Figure 2C and Supplementary Materials Figure S2B). Adenosine treatment promoted BrdU incorporation in C2C12 cell line (Figure 2D,E). Adenosine treatment also induced C2C12 cells to enter synthesis phage during cell cycle, which was determined by flow cytometry after PI staining (Figure 2F and Supplementary Materials Figure S2C). We next sought to determine whether adenosine can regulate C2C12 cell migration. Data from boyden chamber cell migration assay indicated that adenosine distinctly promoted C2C12 migration (Figure 2G,H). Our spheroid assay also confirmed that adenosine enhanced C2C12 migration (Figure 2I,J). Some other genes which regulate cell movement, such as LGALS1 and LGALS3, were activated after adenosine treatment (Figure 2K). Our data suggested that adenosine promotes C2C12 proliferation and migration.

### 2.3. Adenosine Promotes Skeletal Muscle Regeneration Following Acute Injury

**Figure 3:**
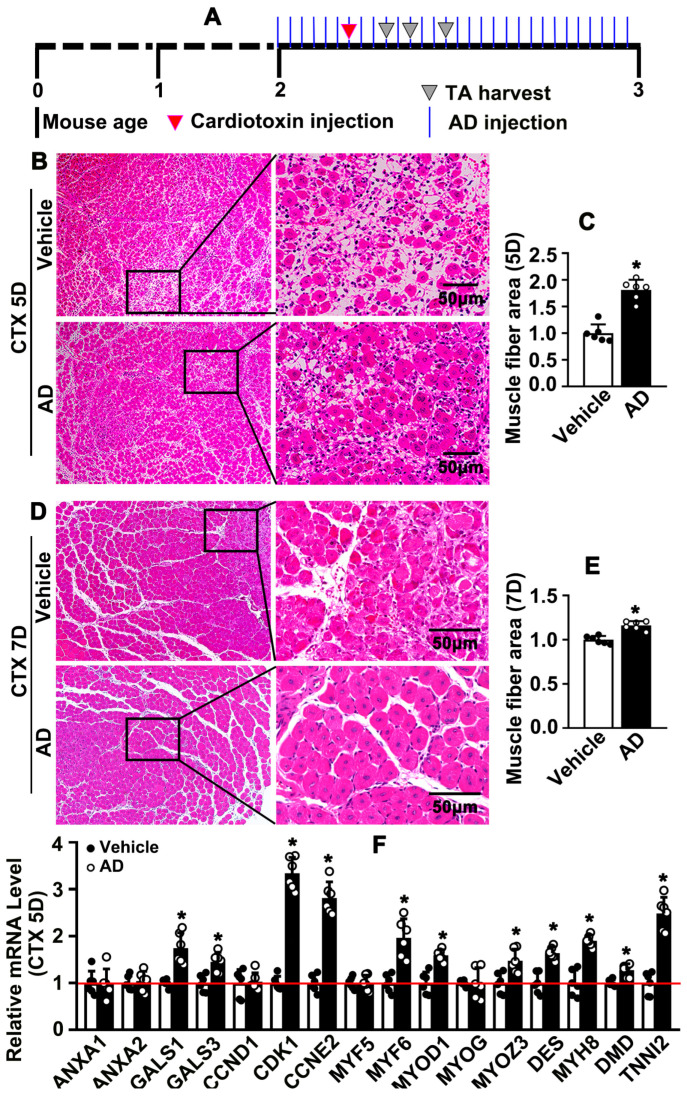
**Adenosine promotes skeletal muscle regeneration following acute injury.** (**A**) Schematic diagram of acute skeletal muscle injury model and adenosine treatment. Two-month-old mice were consecutive intraperitoneal injection with adenosine (10 mg/kg) for 7 days (7D), followed by intramuscular injection of cardiotoxin to induce acute skeletal muscle injury. TA muscles were harvested for further analysis. (**B**) Representative images of H&E staining of TA muscles on day 5 after cardiotoxin injection, and quantification of muscle fibers area were exhibited in (**C**) (*n* = 6, unpaired *t*-test). (**D**) Representative images of H&E staining of TA muscles on day 7 after cardiotoxin injection and muscle fiber areas quantified in (**E**) (*n* = 6, unpaired *t*-test). (**F**) On day 5 after cardiotoxin injection, real time PCR was performed to determine gene expression associated with proliferation, migration and differentiation (*n* = 6, unpaired *t*-test). Data were presented as mean ± SEM, * *p* < 0.05 was considered significant.

To sought to determine whether adenosine is critical for skeletal myofiber maturation, 2-month-old C57BL/6 mice were pretreated with adenosine (10 mg/kg) for 7 consecutive days by intraperitoneal injection. Cardiotoxin intramuscular injection [14] was performed to induce skeletal muscle injury model, which was followed by continued adenosine treatment until tissues were harvested (Figure 3A). Three days after cardiotoxin injection, skeletal muscle myolysis was exhibited in both control group and adenosine treatment group. However, fewer blood cell infiltration was observed after adenosine treatment (Supplementary Materials Figure S3A). Five days after cardiotoxin injection, cardiotoxin-induced skeletal muscle myolysis characterized fragments of myofibers, dissolved or disorganized extracellular matrix, inflammatory cell infiltration, and blood cell infiltration. However, following adenosine treatment, more newly formed myofibers were exhibited. The myofibrils fused with both one another and surviving segments of damaged skeletal myofibers, which were characterized by multiple nuclei located in the center of newly formed myofibers (Supplementary Materials Figure S3B and Figure 3B). The area of newly formed myofibers was increased after adenosine treatment (Figure 3C). The data indicated that adenosine promoted newly formed myofibers and newly fused myofibers. Seven days after cardiotoxin injection, different sizes of newly formed myofibers were exhibited in the vehicle group, with some newly formed myofibers located around the surviving myofiber segment. However, more even sizes of newly formed myofibers, as well as a more appropriate arrangement of the extracellular matrix, were displayed after adenosine treatment (Figure 3D,E). Our data demonstrated that adenosine promotes development of newly formed myofibers. Cardiotoxin can induce skeletal degeneration, and necrosis was exhibited at early injury stage. It is hard to obtain slides at early injury stage for further analysis. However, we performed real-time PCR to determine transcription level of some marker genes that regulate skeletal muscle regeneration. Adenosine enhanced LGALS1, LGALS3, CDK1, CCNE2, MYF6, MYOD1, MYOZ3, MYH8, and TNNI2 transcription 5 days after cardiotoxin injection (Figure 3F). Our data demonstrated that adenosine promotes skeletal muscle regeneration after acute injury.

### 2.4. Adenosine Promotes Differentiation and Fusion of Newly Formed Myofibers

**Figure 4:**
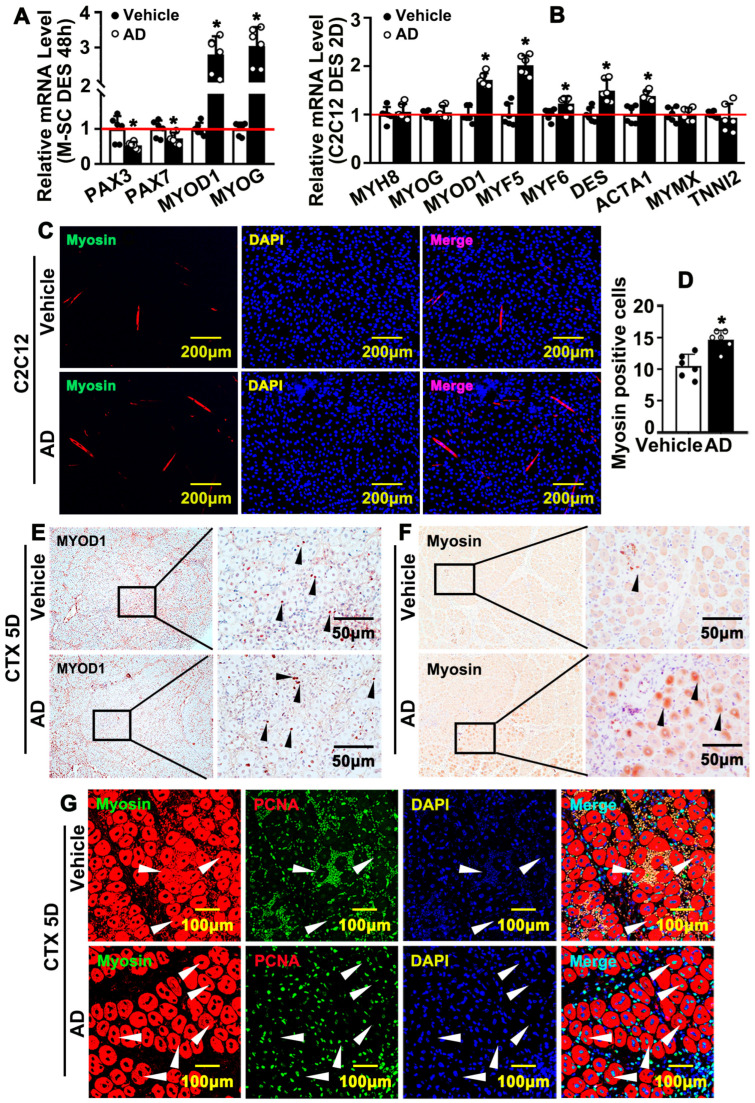
Adenosine promotes differentiation and fusion of newly formed myofibers. (**A**) M-SC differentiation was induced by 2% DES treatment following adenosine treatment for 48 h, and real-time PCR was performed to determine gene transcriptions associated with skeletal muscle differentiation (*n* = 6, unpaired *t*-test). (**B**) C2C12 differentiation was induced by 2% DES treatment in the presence of adenosine for 2 days. Differentiation-related genes of skeletal muscle were evaluated by real-time PCR (*n* = 6; unpaired *t*-test). (**C**) Induction differentiation of C2C12 for 3 days under the existence of adenosine; newly formed myofiber was detected by IF staining against Myosin antibody (scale bar: 100 μM). (**D**) Quantification of Myosin-positive cells (*n* = 6; unpaired *t*-test). (**E**) IHC staining against MYOD1 antibody performed on TA muscle on day 5 after cardiotoxin injection (arrows: positive signals for MYOD1). (**F**) IHC staining against Myosin antibody performed on TA muscle on day 5 after cardiotoxin injection (arrows: positive signals for Myosin). (**G**) IF staining against Myosin and PCNA antibodies on TA muscle slides on day 5 after cardiotoxin injection to visualize the multiple nucleus muscle fibers (White arrows: multiple nucleus muscle fibers). Data were presented as mean ± SEM, * *p* < 0.05 was considered significant.

Unappropriated differentiation and maturation of newly formed myofibers can result in dysfunction of skeletal muscle. We sought to determine whether adenosine can promote myofibers differentiation. murine skeletal muscle satellite cell (M-SC) was treated by 2% DES-containing adenosine for 48 h. Real-time PCR data indicated that transcription levels of PAX3 and PAX7 were significantly decreased, whereas MYOD1 and MYOG dramatically increased (Figure 4A). Differentiation of C2C12 was induced by 2% DES and real-time PCR was performed to evaluate skeletal muscle-differentiated genes. Our data indicated that adenosine enhanced transcription levels of MYOD1, MYF5, MYF6, DES, and ACTA1 (Figure 4B). After DES treatment, IF staining against Myosin antibody was performed. Our data demonstrated that adenosine significantly enhances C2C12 cells’ Myosin expression (Figure 4C,D). We further determined whether adenosine promotes myofiber differentiation in vivo. MYOD1 expression can be induced upon satellite cell activation, and high expression level of MYOD1 is maintained during proliferation phage, and continues into the early differentiated phage. We performed IHC staining on slides for 5 days after cardiotoxin injection to evaluate MYOD1 expression. MYOD1 expression was detectable, whereas MYOD1-expressed cells were increased at this stage (Figure 4E and Supplementary Materials Figure S4A). MYOG is a specific marker that is expressed in differentiated phage. We observed MYOG-positive cells increased after adenosine treatment (Supplementary Materials Figure S4B,C). Following adenosine treatment, expression of Myosin 5 days after cardiotoxin injection increased (Figure 4F and Supplementary Materials Figure S4D). PCNA-positive cells were shown at this stage, and more multiple nuclei within newly formed myofibers were exhibited after adenosine treatment (Figure 4G and Supplementary Materials Figure S4E,F). The date demonstrated that adenosine promotes differentiation and fusion of newly formed myofibers.

### 2.5. Adenosine Dynamically Distributes Between Extracellular and Intracellular, Which Triggers an ADK-Dependent Intracellular Signaling Pathway

Adenosine, an endogenous purine nucleoside, is critical in regulating multiple physiological processes. However, whether adenosine regulates skeletal muscle adenosine kinase expression and whether ADK is required during skeletal muscle regeneration are largely unknown.

**Figure 5:**
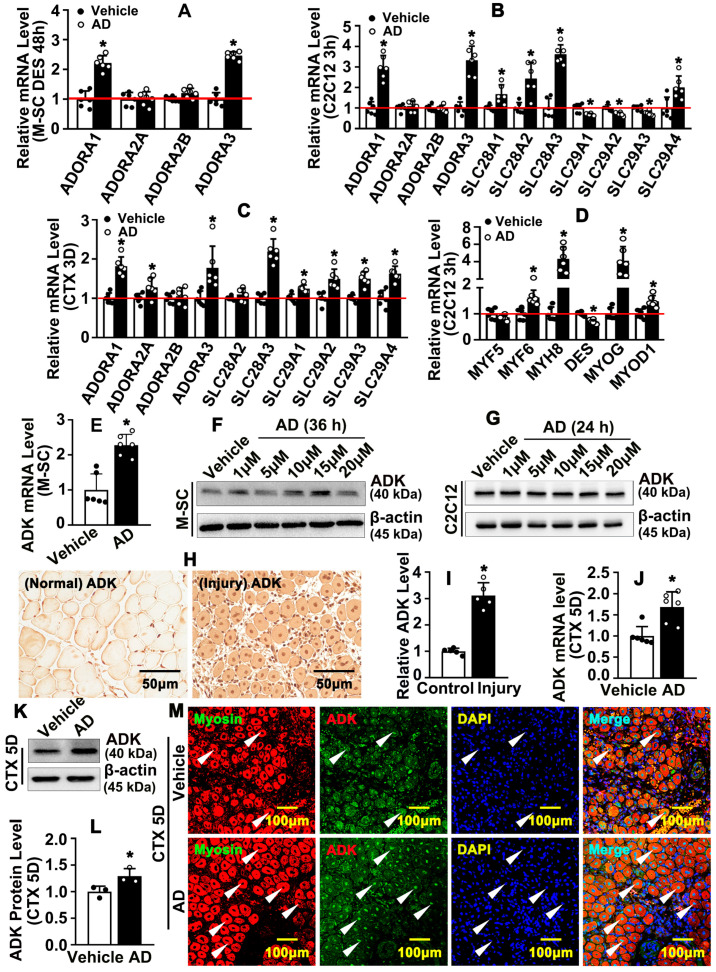
**Adenosine dynamically distributes between extracellular and intracellular, which triggers an ADK-dependent intracellular signaling pathway.** (**A**) Real-time PCR was performed on M-SC to determine transcription levels of adenosine receptors after adenosine treatment for 48 h (*n* = 6, unpaired *t*-test). (**B**) C2C12 cells were treated with adenosine (10 μM) for 3 h, and transcription levels of adenosine receptors and nucleoside transporters were determined by real-time PCR (*n* = 6, unpaired *t*-test). (**C**) Transcription levels of adenosine receptors and nucleoside transporters were detected by real-time PCR on day 3 after cardiotoxin injection (*n* = 6; unpaired *t*-test). (**D**) C2C12 cells were treated with adenosine for 3 h and transcription levels of differentiation-related genes were evaluated by real-time PCR (*n* = 6; unpaired *t*-test). (**E**) Real-time PCR was performed to determine ADK transcription level in M-SC after adenosine treatment for 30 h (*n* = 6; unpaired *t*-test). (**F**) M-SCs were treated by different doses of adenosine for 36 h, and Western blotting was performed to evaluate ADK protein level. (**G**) C2C12s were treated by different doses of adenosine for 24 h, and Western blotting was performed to evaluate ADK protein level. (**H**) Acute injury of skeletal muscle induced by cardiotoxin injection; IHC staining against ADK antibody performed on TA muscle on day 5 after cardiotoxin injection. The relative expression of ADK was quantified in (**I**) (*n* = 5; unpaired *t*-test). (**J**) ADK transcription level in TA muscle on day 5 after cardiotoxin injection was determined by real-time PCR (*n* = 6; unpaired *t*-test). (**K**) Western blotting was performed to determine ADK protein level in TA muscle on day 5 after cardiotoxin injection, and ADK protein level was quantified in (**L**) (*n* = 3; unpaired *t*-test). (**M**) IF staining against ADK and Myosin antibodies was performed on TA muscle on day 5 after cardiotoxin injection; newly formed myofibers were visualized by staining with Myosin antibody (arrows: ADK positive muscle fibers). Quantitative data were presented as mean ± SEM, * *p* < 0.05 was considered significant.

We treated satellite cells with adenosine, and real-time PCR performed to determine transcription levels of adenosine receptors. Our results indicated that different time points of adenosine treatment significantly induced ADORA1 and ADORA3 transcription (Figure 5A and Supplementary Materials Figure S5A). We treated C2C12 with adenosine, and real-time PCR was performed to determine transcription levels of concentrative nucleoside transporters and equilibrative nucleoside transporters. Results indicated that transcription levels of concentrative nucleoside transporter SLC28A1, SLC28A2, and SLC28A3, as well as equilibrative nucleoside transporter SLC29A4, were increased (Figure 5B). Our results indicated that different time points of adenosine treatment, including 1 h, 6 h, and 12 h, dramatically induced ADORA1 and ADORA3 transcription levels, whereas ADORA2A and ADORA2B were suppressed. Adenosine treatment for 1 h, 6 h, and 12 h significantly enhanced SLC28A1, SLC28A2, and SLC28A3 transcription levels (Supplementary Materials Figure S5B–D). Similar studies were performed on animal models; at 3 days after cardiotoxin injection, adenosine treatment dramatically enhanced transcription of ADORA1, ADORA3, SLC28A3, and SLC29A4 (Figure 5C). Five days after cardiotoxin injection, high transcription levels of ADORA1, SLC28A3, and SLC29A4 were exhibited after adenosine treatment (Supplementary Materials Figure S5E). Adenosine treatment significant enhanced MYF6, MYH8, MYOG, and MYOD1 transcription levels in C2C12 (Figure 5D). Our results indicated that adenosine was dynamically distributed between extracellular and intracellular through concentrative nucleoside transporter and equilibrative nucleoside transporters, which was associated with skeletal muscles maturation.

To determine whether dynamic distribution of adenosine can trigger multiple intracellular signaling pathways, we treated satellite cells with adenosine for 24 h. Our real-time PCR data indicated that the Notch signaling pathway, HIF signaling pathway, as well as inflammatory-related signaling pathway were involved in adenosine treatment, especially in dramatically induced transcription levels of ADK and PFKFB3 (Supplementary Materials Figure S6A). Our data indicated that dynamic distribution of adenosine can trigger multiple intracellular signaling pathways.

Our previously study demonstrated that enhanced expression of ADK dramatically decreased endothelial cell adenosine concentration, which play a critical role in attenuating vascular inflammation [33]. However, whether adenosine regulates skeletal muscle ADK expression and whether ADK is required during skeletal muscle regeneration, are largely unknown. Adenosine treatment induced ADK transcription level in satellite cell, which was evaluated by real-time PCR (Figure 5E). Different doses of adenosine treatment and different time points of adenosine treatment induced ADK protein level in satellite cells (Figure 5F and Supplementary Materials Figure S6B–D). Adenosine treatment also induced ADK transcription level in C2C12 (Supplementary Figure S6E). Following different doses of adenosine treatment and different time points of treatment, ADK expression level was significantly increased in C2C12 (Figure 5G and Supplementary Materials Figure S6F–H). In order to determine whether injury itself induced ADK expression. We performed IHC staining against ADK antibody after cardiotoxin intramuscular injection. Our results indicated that ADK expression was dramatically increased after cardiotoxin intramuscular injection (Figure 5H,I). However, whether increased expression of ADK after skeletal muscle injury was associated with adenosine treatment is ambiguous. To better understand whether adenosine regulates ADK expression during skeletal muscle regeneration, we harvested injury tibialis anterior muscles 5 days after cardiotoxin injection, and evaluated ADK transcription level and protein level. Our data indicates that adenosine treatment dramatically promoted both transcription level and protein level of ADK in injured skeletal muscle (Figure 5J–L). We also determined ADK expression after cardiotoxin injection by IHC staining and IF staining against ADK antibody. Higher protein level of ADK was exhibited within newly formed myofibers, especially those located in the nucleus (Figure 5M and Supplementary Materials Figure S6I,J). Our data indicated that adenosine induces ADK expression during skeletal muscle regeneration.

### 2.6. Adenosine Promotes PFKFB3-Mediated Glycolysis Metabolism During Skeletal Muscle Regeneration

**Figure 6:**
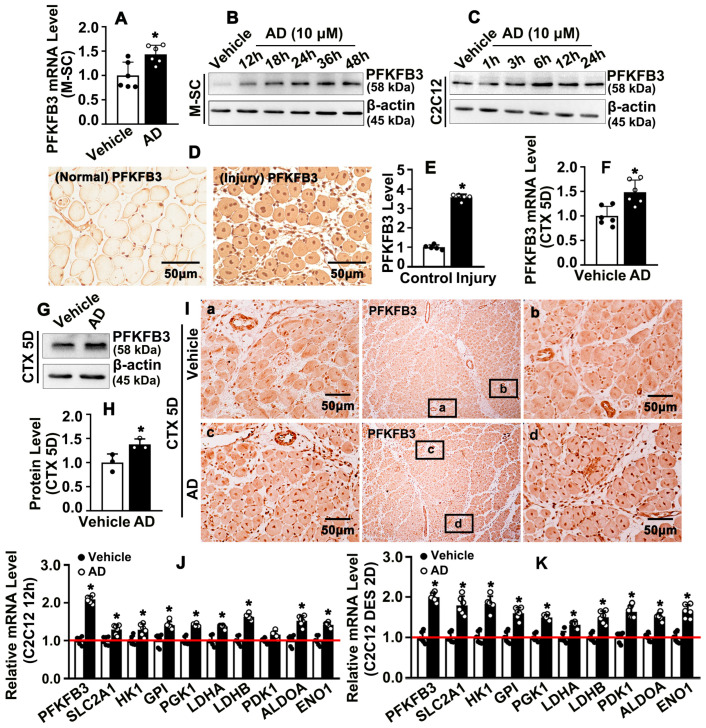
**Adenosine promotes PFKFB3-mediated glycolysis metabolism during skeletal muscle regeneration.** (**A**) PFKFB3 transcription level in M-SC was detected by real-time PCR after adenosine (10 μM) treated for 30 h (*n* = 6; unpaired *t*-test). (**B**) M-SCs were treated by adenosine, protein was harvested at different time points, and PFKFB3 expression was determined by Western blotting. (**C**) C2C12s were treated by adenosine, protein was harvested at different time points, and PFKFB3 expression was determined by Western blotting. (**D**) Acute injury of skeletal muscle was induced by cardiotoxin injection; IHC staining against PFKFB3 antibody on TA muscle on day 5 after cardiotoxin injection. The relative expression of PFKFB3 was quantified in (**E**) (*n* = 5; unpaired *t*-test). (**F**) Transcription level of PFKFB3 in TA muscle on day 5 after cardiotoxin injection was evaluated by real-time PCR (*n* = 6; unpaired *t*-test). (**G**) PFKFB3 protein level in TA muscle on day 5 after cardiotoxin injection was examined by Western blotting. The protein level of PFKFB3 was quantified in (**H**) (*n* = 3; unpaired *t*-test). (**I**) IHC staining against PFKFB3 was performed on TA muscle on day 5 after cardiotoxin injection. (**J**) C2C12 was treated with adenosine (10 μM) for 12 h, and real-time PCR was performed to evaluate glucose metabolism related genes (*n* = 6; unpaired *t*-test). (**K**) Differentiation of C2C12 was induced by DES treatment for 2 days following existence of adenosine, and real-time PCR performed to evaluate glucose metabolism-related genes (*n* = 6; unpaired *t*-test). Quantitative data were presented as mean ± SEM, * *p* < 0.05 was considered significant.

Adenosine treatment also obviously increased expression of PFKFB3 in C2C12. Our previously publication indicated that PFKFB3 is critical in regulating metabolism of glucose [40]. Whether adenosine can regulate skeletal muscle glucose metabolism is largely undefined. We sought to determine whether adenosine can regulate PFKFB3 expression during skeletal muscle regeneration. Adenosine treatment significant induced PFKFB3 transcription level in satellite cell (Figure 6A). Different time points or different doses of adenosine treatment dramatically enhanced PFKFB3 expression (Figure 6B and Supplementary Materials Figure S7A–C). We treated C2C12 with different doses of adenosine, and observed that doses ranged from 1μM to 20μM can enhance PFKFB3 protein level (Supplementary Materials Figure S7D,E). Different time points of adenosine treatment also induced PFKFB3 protein level (Figure 6C and Supplementary Materials Figure S7F). In order to determine whether injury itself induced PFKFB3 expression, we performed IHC staining against PFKFB3 antibody after cardiotoxin intramuscular injection. Our data indicated that a very low protein level of PFKFB3 was observed in mature skeletal muscle, whereas the expression level of PFKFB3 was tremendously induced during skeletal muscle regeneration (Figure 6D,E). We harvested injured skeletal muscle 5 days after cardiotoxin injection, and observed that adenosine treatment significantly enhanced both PFKFB3 transcription and PFKFB3 protein level (Figure 6F–H). IHC staining against PFKFB3 antibody indicated that adenosine treatment significantly enhanced PFKFB3 expression within newly formed myofibers, as well as within some other cell types 5 days after cardiotoxin injection (Figure 6I and Supplementary Materials Figure S7G,H). Those data indicated that adenosine promotes PFKFB3 expression during skeletal muscle regeneration.

Since PFKFB3 is a key enzyme, it plays a critical role in regulating glucose metabolism. We treated C2C12 with adenosine under normal culture conditions, and our real-time PCR data indicated that adenosine promoted expression of enzymes that regulate glucose metabolism, such as PFKFB3, SLC2A1, HK1, GPI, PGK1, LDHB, ALDOA, and ENO1, and PFKFB3 was tremendously induced after adenosine treatment (Figure 6J). Seminary results were exhibited in differentiating C2C12, which was induced by DES (Figure 6K). Our data indicated that adenosine promotes PFKFB3-mediated glucose metabolism during skeletal muscle regeneration.

### 2.7. ADK Interacts with PFKFB3 During Skeletal Muscle Regeneration

**Figure 7:**
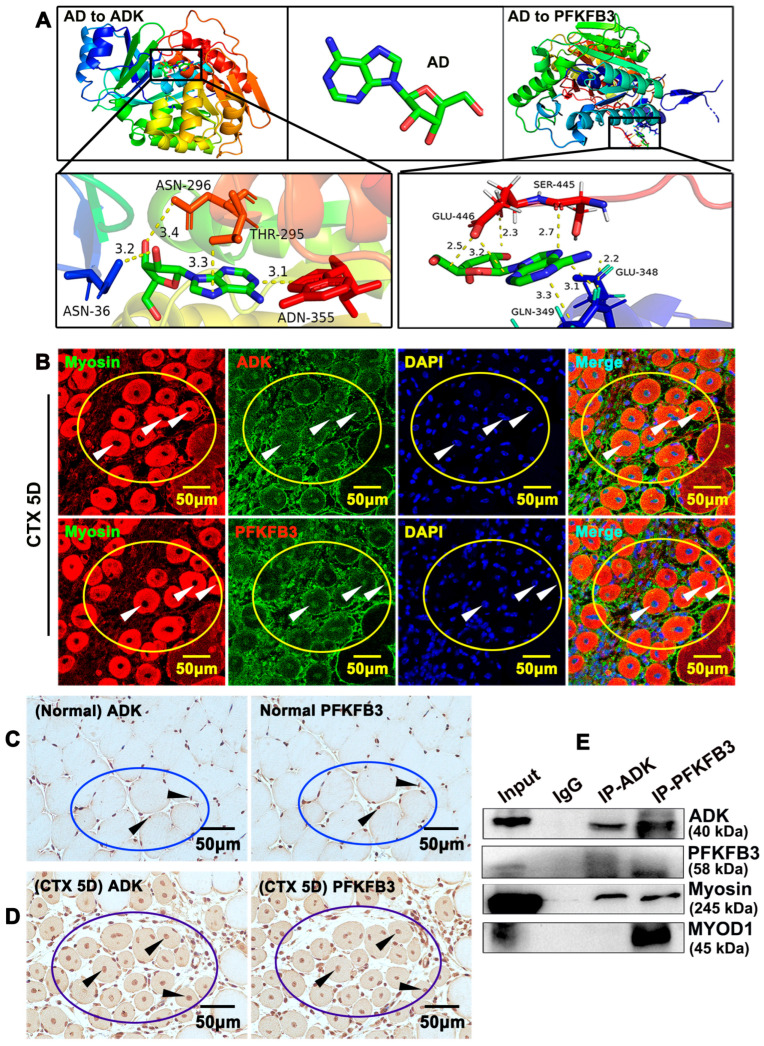
**ADK interacts with PFKFB3 during skeletal muscle regeneration.** (**A**) Interactions between AD with ADK and PFKFB3 were determined by molecular docking simulation. The three-dimensional structures of ADK and PFKFB3 were obtained from the RCSBPDB database. The protein structures of ADK (1bx4) and PFKFB3 (6hvi) served as receptors, and adenosine was used as a small-molecule ligand to determine the docking sites between receptors and ligands. When the binding energy value is less than zero, these proteins are considered to spontaneously bind and interact with each other. Serial sectioning was performed, and adjacent sections were collected to detect different genes expressed in the same cells. (**B**) Adjacent serially sectioned slides were collected from TA muscle on day 5 after cardiotoxin injection. IF staining against ADK and PFKFB3 antibodies on adjacent slides was performed to determine expression of ADK and PFKFB3 in the same newly formed myofibrils. (**C**) Adjacent serially sectioned slides were collected, and IHC staining against ADK and PFKFB3 antibodies on adjacent slides was performed to determine whether ADK and PFKFB3 expressed in the same myofibers. (**D**) Adjacent serially slides were collected on day 5 after cardiotoxin injection, and IHC staining against ADK and PFKFB3 antibodies on adjacent slides was performed to determine whether ADK and PFKFB3 expressed in the same myofibers (Circles: Same cell population in serial sections. arrows: ADK- or PFKFB3-positive signaling). (**E**) Total protein was extracted from TA muscles after 5 days of cardiotoxin injection using RIPA lysis buffer, and CO-IP was performed to determine protein interaction. Cell lysates were precleared with anti-species-specific IgG beads. The precleared cell lysate was incubated with PFKFB3 and ADK antibodies for 3 h at 4 °C, following incubation with pre-equilibrated protein A/G agarose beads. The targets of CO-IP were evaluated by Western blotting.

Whether ADK regulates PFKFB3 expression during skeletal muscle regeneration has never been studied. We sought to analyze molecular docking simulation of adenosine with ADK and PFKFB3, which is determined by the binding energy using Autodock Vina 1.5.6 software developed by Olson’s research group. The three-dimensional structures of ADK and PFKFB3 were obtained from the RCSBPDB database (http://www.rcsb.org/ accessed on 14 December 2025). Multiple potential binding areas of adenosine with ADK and PFKFB3 were exhibited (Figure 7A). When a value of binding energy is less than zero, those proteins were considered spontaneously binding and interacting with each other. Approximately −7.2 of binding energy exhibited between adenosine and ADK, and −6.6 of binding energy exhibited between adenosine and PFKFB3 (Supplementary Materials Figure S8A).

Consecutive serial sectioning was performed, and adjacent sections were collected to detect different genes expressed in the same cells. We collected adjacent serially sectioned slides to detect ADK and PFKFB3 expression separately within the same cells. The IF staining indicated both ADK and PFKFB3 were expressed ubiquitously within newly formed myofibers, including member, cytoplasm, and nucleus (Figure 7B). Very low expression levels of ADK and PFKFB3 were identified in adult skeletal muscle and majority of expression was restricted to nucleus, which can be detected using IHC staining (Figure 7C). However, ADK and PFKFB3 expression was remarkably induced in newly formed myofibers and some other cell types following injury stress, which was exhibited ubiquitously within newly formed myofibers, such as member cytoplasm and nucleus. Using consecutive paraffin-embedded slides, the IHC staining indicated that ADK and PFKFB3 can be expressed within the same cells (Figure 7D). Those data suggested that both ADK and PFKFB3 expressed in newly formed myofibers, and both ADK and PFKFB3 potentially can bind to adenosine. To confirm this hypothesis, we performed a CO-immunoprecipitation assay to evaluate interaction between ADK and PFKFB3. Using protein prepared from C2C12, we observed that ADK can bind to PFKFB3 (Supplementary Materials Figure S8B). Protein from tibialis anterior muscle after cardiotoxin injection demonstrated that ADK and PFKFB3 can bind to each other as well as to Myosin. However, only PFKFB3 can bind to MYOD1 (Figure 7E). Those data demonstrated that adenosine kinase interacts with PFKFB3 during skeletal muscle regeneration.

### 2.8. Interruption of the Interaction Between ADK-Dependent Intracellular Signaling Pathway and PFKFB3-Mediated Glycolytic Metabolism Can Attenuate Adenosine-Induced Skeletal Muscle Regeneration

**Figure 8:**
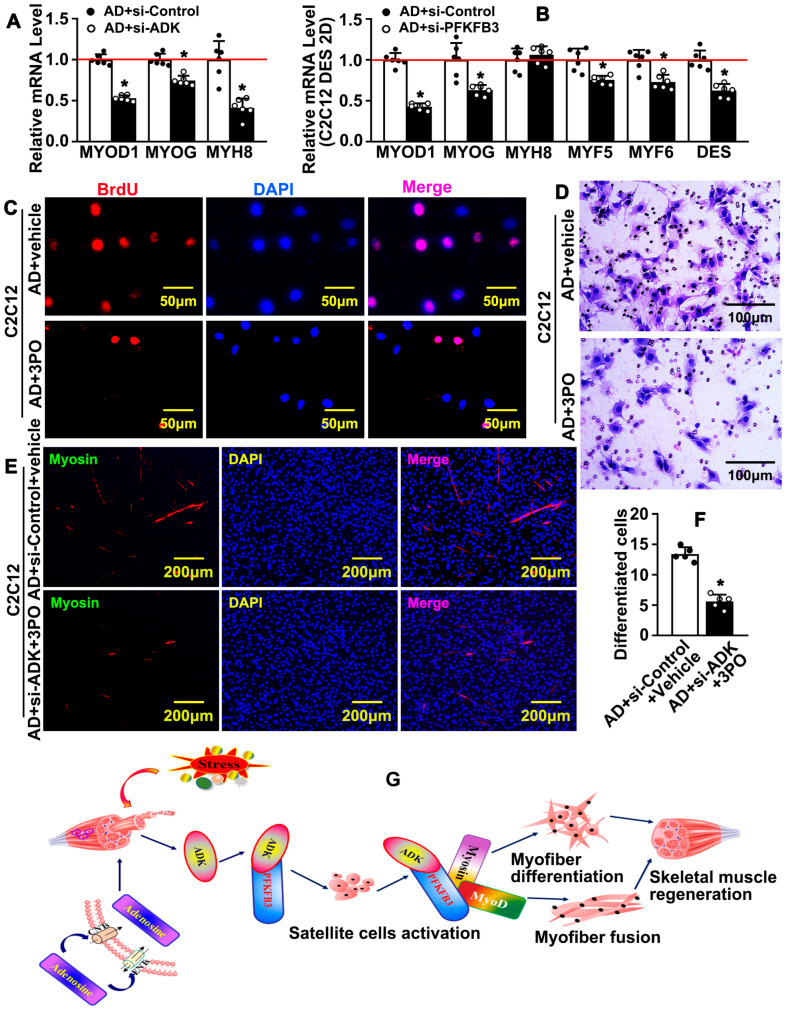
Interruption of the interaction between ADK-dependent intracellular signaling pathway and PFKFB3-mediated glycolytic metabolism can attenuate adenosine-induced skeletal muscle regeneration. (**A**) C2C12 cells were transfected with scrambled siRNA and siRNAs targeting murine ADK, following adenosine treatment for 48 h, and real-time PCR was performed to determine transcription levels of MYOD1, MYOG, and MYH8 (*n* = 6; unpaired *t*-test). (**B**) C2C12 cells were transfected with scrambled siRNA and siRNAs targeting murine PFKFB3, following adenosine treatment, and real-time PCR was performed to evaluate transcription levels of myofiber differentiation-related genes (*n* = 6; unpaired *t*-test). (**C**) After treatment with 3PO (20 μM), following adenosine (10 μM) treatment for 12 h and incubation with BrdU labeling reagent for 6 h, IF staining was performed to determine BrdU incorporation. (**D**) After treatment with 3PO (20 μM), following adenosine (10 μM) treatment, boyden chamber cell migration assay was performed to determine the migration of C2C12 cells. (**E**) After transfection of C2C12 cells with scrambled siRNA and siRNAs targeting murine ADK, cell differentiation was induced by DES-containing 3PO (20 μM) and adenosine (10 μM) for 3 days, and IF staining against Myosin antibody was performed to determine fusion of myofibers. (**F**) Quantification of Myosin-positive C2C12 cells in E (*n* = 5, unpaired *t*-test). Quantitative data are presented as mean ± SEM, * *p* < 0.05 was considered significant. (**G**) Schematic diagram demonstrated that adenosine dynamically distributes between extracellular and intracellular, and rapidly induces ADK-associated intracellular signaling pathway, which interacts with PFKFB3-mediated glycolytic metabolism to promote satellite cell activity, as well as new myofiber formation, differentiation, and fusion, and eventually promotes skeletal muscle regeneration after injury stress.

To determine whether adenosine triggers an ADK-dependent intracellular signaling pathway’s interaction with PFKFB3-mediated glycolytic metabolism to promote newly formed myofibers development, we silenced ADK or PFKFB3 in C2C12 by transfection with scrambled siRNA and siRNAs targeting murine ADK or PFKFB3 (Supplementary Materials Figure S8C). We silenced ADK in C2C12 by transfection with siRNAs targeting murine ADK using Lipofectamin 2000 reagent. Real-time PCR was performed to validate ADK knockdown efficiency. Three siRNAs have been synthesized (S1, S2 and S3) and S2 have been chosen for further study (Supplementary Materials Figure S8D). Knockdown of ADK in C2C12 suppressed transcription of MYOD1, MYOG, and MYH8 (Supplementary Materials Figure S8E). Knockdown of ADK-attenuated adenosine treatment induced MYOD1, MYOG, and MYH8 transcription (Figure 8A). We also silenced PFKFB3 in C2C12 by transfection with scrambled siRNA and siRNAs targeting murine PFKFB3. Real-time PCR was performed to validate PFKFB3 knockdown efficiency (Supplementary Materials Figure S8F). Three siRNAs have been synthesized (S1, S2, and S3), and S2 has been chosen for further study. Knockdown of PFKFB3 in C2C12 inhibited transcription of MYOD1, MYOG, MYF8, and MYF6 (Supplementary Materials Figure S8G). Knockdown of PFKFB3 dramatically attenuated adenosine treatment-induced transcriptions of MYOD1, MYOG, MYF5, MYF6, and DES (Figure 8B). We further treated C2C12 with 3PO, a PFKFB3 inhibitor. 3PO treatment significant suppressed MYOD1 expression. 3PO treatment dramatically inhibited adenosine-induced MYOD1 expression (Supplementary Materials Figure S9A,B). BrdU incorporation assay was performed, and we observed that 3PO treatment attenuated adenosine treatment-induced BrdU incorporation (Figure 8C and Supplementary Materials Figure S9C). We performed boyden chamber cell migration assay and observed that 3PO treatment attenuated adenosine treatment-induced cell migration (Figure 8D and Supplementary Materials Figure S9D). We performed a knockdown of ADK by siRNA transfection, as well as inhibition of PFKFB3 by 3PO treatment. Inhibition of ADK/PFKFB3 signaling pathway dramatically attenuated adenosine-induced differentiation and fusion of myofibers (Figure 8E,F and Supplementary Materials Figure S9E).

## 3. Discussion

We provided evidence that adenosine triggers intracellular signaling pathway, which is critical for skeletal muscle regeneration. Multiple stresses contribute to myopathy development, including hypoxia condition [41], immune function impairment [42], endocrine disorder, metabolic disorder, and neurological disfunction. Short half-life of adenosine exhibited was due to multiple enzymes associated with catalytic reactions [30]. Some studies indicated that increased metabolic disorder, hypoxia condition, and tissue injury can cause increased adenosine concentration [43,44]. Our studies indicated that adenosine treatment induced concentrative nucleoside transporter and equilibrative nucleoside transporter transcriptions, which suggested that adenosine can be dynamically distributed between intracellular and extracellular in myofibers.

Our studies demonstrated that adenosine triggers an ADK-dependent intracellular signaling pathway interaction with PFKFB3-mediated glycolytic metabolism to promote newly formed myofibers development. Knockdown of ADK in C2C12 dramatically decreased PFKFB3 transcription. However, knockdown of PFKFB3 in C2C12 did not change transcription of ADK (Supplementary Materials Figure S9F,G). The regeneration process of skeletal muscle is so complicated that some other signaling pathways, including NOTCH, HIF, FGF, PI3K/Akt/mTOR, Wnt/ꞵ-catenin, TGF-ꞵ, MAPK, PFKFB3 [45], and adenosine [46] signaling pathways, contribute to skeletal muscle regeneration.

### 3.1. Multiple Cell Types Are Involved During Skeletal Muscle Regeneration

Adult skeletal muscle is composed of a group of fascicules of multinucleated myofibers, and myonuclear are located at the periphery of myofibers. The quiescent satellite cell was well studied and was critical for skeletal muscle regeneration.

It is hard to identify or distinguish satellite cells from other cell types within adult skeletal muscle. Our studies indicated that macrophage was identified at an early stage after skeletal muscle injury [47], and endothelial intracellular adenosine contributed to vascular inflammation regulation [33]. We did observe that adenosine treatment induced macrophage infiltration at early injury stage (Supplementary Materials Figure S10A–C). Adenosine treatment also regulated inflammatory response (Supplementary Materials Figure S10D,E). We focused on satellite cells activation, proliferation, differentiation, and newly formed myofiber fusion, which were regulated by adenosine treatment. However, some other cell types, such as fibroblast cells, adipocyte, endothelial cell, smooth muscle cells, neurocyte, are indispensable during skeletal muscle regeneration. Intracellular adenosine was reported to suppress T cells in cancer development [48]. Whether adenosine regulated skeletal muscle regeneration through inflammatory signaling pathways will need further studies.

### 3.2. Some Other Mechanisms Besides Bioenergy Supplements Are Involved in Skeletal Muscle Regeneration

Metabolism of adenosine leads us to generate ATP. This catalytic reaction was regulated by ADK [22]. Our previous studies indicated that inhibition of ADK resulted in increased intracellular adenosine concentration [32]. PFKFB3 is a critical enzyme in regulating glucose metabolism to generate ATP and multiple intermediate metabolites [40,49]. In this study, we identified that ADK induced by adenosine after skeletal muscle injury can enhance PFKFB3 expression and interacts with PFKFB3. However, this interaction between ADK and PFKFB3 cannot be understood via the synergistic affection. Both ADK and PFKFB3 can promote ATP generation and this synergistic affection, if it really exists, is against kinetic theory energy. There must be some other mechanisms beyond bioenergy that are involved in skeletal muscle regeneration.

### 3.3. Adenosine-Triggered Intracellular Signaling Pathway Can Affect Multiple Skeletal Markers During Skeletal Muscle Regeneration

Multiple pathological processes induced after skeletal muscle injury stress, including tissue degeneration, quiescent satellite cells activation, satellite cell differentiation, newly formed myofiber fusion and skeletal muscle remodeling, are regulated by different signaling pathways. Most specific genes are transiently expressed during specific phases. Each pathological process is tightly related to one other, and it is hard to distinguish those processes in both animal model and cell culture conditions. To fully understand the function of adenosine-induced intracellular ADK/PFKFB3 signaling pathways on skeletal muscle regeneration, skeletal muscle ADK knockout mice combined with clinical research are also indispensable.

Adenosine triggers an intracellular signaling pathway that is critical for skeletal muscle regeneration. However, while cardiotoxin injection can induce acute skeletal muscle injury, it cannot recapitulate the pathological progression of myopathy. The underlying mechanisms of myopathy require further investigation using tissue-specific knockout mouse models combined with clinical observations.

## 4. Materials and Methods

### 4.1. Murine Skeletal Satellite Cell Culture

Isolation of M-SC is based on our previous publication [47]. In brief, skeletal muscles, including tibialis anterior (TA), gastrocnemius, and quadriceps muscles, were collected from 4-week-old C57BL/6 mice. Skeletal muscle tissues were cut into small pulps and, following digestion in a collagenase solution, we centrifuged and discarded the supernatant. We cultured the cells for 2 h, transferred the supernatant unattached cells, and cultured them again. Similar processes were performed until passage 4.

### 4.2. C2C12 Cell Line Culture and Differentiation Induction

C2C12 cell line was purchased from ATCC (CRL-1772, Manassas, VA, USA) and cultured in DMEM medium containing 10% fetal bovine serum (FBS, Gibco, Grand Island, NY, USA). Differentiation of C2C12 was induced by 2% Donor Equine Serum (DES, Ramat Gan, Israel) incubation.

### 4.3. CCK8 Cell Proliferation Assay

3 × 10 ^3^ C2C12 cells or Murine satellite cells were seeded in a 96-well culture plate, treated with adenosine (10 μΜ, Sigma, St. Louis, MO, USA) for 24 h, and the absorbance at 450 nm was evaluated using the CCK-8 kit (AbMole, Houston, TX, USA).

### 4.4. BrdU Incorporation Assay

The mouse C2C12 or murine satellite cells were suspended in adenosine (10 μM) medium, cultured with BrdU labeling reagent (Sigma) for 6–12 h. IF staining was performed to determine the BrdU-incorporated cells. For BrdU (Cell Signaling Technology, Danvers, MA, USA), following staining, DNA denaturization using 2N HCl, and antibodies incubation, images were captured using confocal microscopy (LS510, Zeiss, Oberkochen, Germany).

### 4.5. Spheroid Sprouting Assay

The spheroid sprouting assay was performed as described previously [50]. Methylcellulose solution (Sigma) was prepared by dissolving 6 g methylcellulose (sigma) in 250 mL prewarmed serum free medium, and 250 mL DMEM containing 10% serum was added. We suspended cells to be dissolved in methylcellulose solution, which we prepared by adding 10 mL methylcellulose solution and 40 mL culture medium to form the spheres. We added neutralized collagen solution to a 24-well culture plate and incubated them at 37 °C until the collagen solidified. We mixed spheres with dissolved collagen solution and transferred to the collagen-solidified culture plate. We solidified the culture plate for 30 min at 37 °C. We added 200 μL of complete medium containing adenosine and cultured them overnight. The spheroid sprouting was visualized after calcein AM (Beyotime, Shanghai, China) staining. Images were captured using confocal microscope (Leica Microsystem CMS GmbH, Mannheim, Germany), and the number of sproutings and the sprouting length of each sphere were analyzed by Image J software 1.46r.

### 4.6. Boyden Chamber Migration Assay

1 × 10^6^ murine satellite cells or C2C12 cells were suspended in 100 ul FBS free culture media and seeded in a Boyden Chamber (FALCON, Corning, NY, USA). We set up the Boyden Chamber into a 24-well culture plate that contained 500 μL complete culture medium (10% FBS) and 5 μM adenosine. We fixed the cells after incubation for 12 to 24 h, manually counting cells numbers after crystal violet staining [51].

### 4.7. Induction of Acute Skeletal Muscle Injury by Cardiotoxin Intramuscular Injection

We performed cardiotoxin intramuscular injection to induce acute skeletal muscle injury as in the previous publication [14,47]. Briefly, 8-week-old C57BL/6 mice were pretreated with adenosine (10 mg/kg) for 7 consecutive days by intraperitoneal injection [52,53]. The animals were anesthetized with ketamine (80 mg/kg) and xylazine (5 mg/kg) by intraperitoneal injection. The skin over the TA muscle was shaved and sterilized with tincture of iodine. Approximately 10 μM cardiotoxin (BOYAO, Shanghai, China) working solution was prepared with sterilized phosphate-buffered saline, and 20 μL cardiotoxin working solution was intramuscularly injected in the TA muscle [14]. The animal was consecutively treated with adenosine. The animals were sacrificed using CO2, and the TA muscles were harvested at different time points and underwent paraffin embedded for further analysis.

### 4.8. Hematoxylin and Eosin (H&E) Staining, Immunohistochemistry (IHC), and Immunofluorescence Staining (IF)

We harvested murine TA muscles at different time points, fixed them with 4% paraformaldehyde solution at 4 °C with paraffin embedded, and we collected slides of 5 μm thickness. H&E staining was performed as in our previous study [54]. For IHC staining, the slides were deparaffinized, and antigen retrieval was performed by citric acid treatment. After antigenic unmasking (Vector Laboratories, Newark, CA, USA), they were incubated with primary antibody at 4 °C, followed by incubation with biotinylated secondary antibody for 1 h (Vector Laboratories, USA), and incubated with ABC solution (Vector Laboratories, Newark, CA, USA) for 30 min. The special targets were visualized after DAB solution was added (Vector Laboratories, USA). For IF staining, the deparaffinized slides, permeabilized with PBS, contained 0.25% Triton-X-100, blocked with 10% goat serum, incubated with primary antibodies at 4 °C, and incubated with Alexa 594-conjugated or Alexa 488-conjugated secondary antibody for 1 h. Nuclei were visualized with 4’, 6’-diamidino-2-phenylindole (DAPI) staining. For BrdU staining, DNA was denaturized using 2N HCl and followed by antibodies incubation. Images were captured using confocal microscopy (LS510, Zeiss, Oberkochen, Germany).

Antibodies used in this study included PCNA (Cell Signaling Technology), MKI67 (Cell Signaling Technology), PAX7 (NOVUS, Chesterfield, MI, USA), MYOD1 (NOVUS, Chesterfield, MI, USA), Myosin (Sigma), MYOG (Invitrogen, Carlsbad, CA, USA), ADK (Invitrogen), and PFKFB3 (Proteintech, Rosemont, IL, USA).

### 4.9. Real-Time PCR Analysis

Total RNA from murine satellite cells, C2C12 cells, and murine tibialis tissues were extracted using Trizol reagent (Thermo Invitrogen, Waltham, MA, USA). Quantity and quality of RNA were monitored by a spectrophotometer (Denovix, Wilmington, DE, USA). Approximately 600 ng RNA was used as a template, and random hexamer primers were used for reverse transcription reaction to obtain cDNA using iScript cDNA synthesis kit (Takara Bio. Inc. Kusatsu, Shiga, Japan). Real-time PCR performed duplication for each sample on the Bio-Rad Real-Time PCR system (CFX Maestro 1.1, Bio-Rad, Hercules, CA, USA). The relative gene transcription levels were analyzed using the 2^−ΔΔct^ method.

### 4.10. siRNA Transfection

Scrambled siRNA (si-Control) and siRNAs targeting murine ADK or PFKFB3 were synthesized form GenePharma (Shanghai, China). The siRNAs were transfected into murine C2C12 by using Lipofectamin 2000 reagent (GenePharma, Shanghai, China).

### 4.11. Protein Extraction and Western Blotting Analysis

Protein from M-SC, C2C12 cells and murine tibialis tissues extracted using RIPA lysis buffer (Biosharp Life Sciences, Beijing. China). Protein concentration was determined by using BCA kit (Biosharp, Nanjing, China). Protein was denatured at 98 °C, separated by sodium dodecyl sulfate–polyacrylamide gel electrophoresis (SDS-PAGE), and transferred onto polyvinylidene fluoride membranes. Afterwards, we blocked them with 5% fat free milk, and incubated them with specific antibodies. Images were captured using Image Quan LAS4000 Image Station and quantified densities of protein bands using Image J software 1.46r.

### 4.12. Protein-to-Protein Interaction (PPI) Network Analysis

The interaction between genes, which are critical in regulating skeletal muscle regeneration, were analyzed using STRING database (https://www.string-db.org/ accessed on 14 December 2025). The diagram of PPI was generated by Cytoscape 3.8.2 software.

### 4.13. Molecular Docking Simulation of Adenosine with ADK and PFKFB3

A molecular docking simulation was performed to evaluate the binding energy of adenosine with ADK and PFKFB3 using Autodock Vina 1.5.6 software, which was developed by Olson’s research group [55]. The three-dimensional structures of ADK and PFKFB3 were obtained from the RCSBPDB database (https://www.rcsb.org accessed on 14 December 2025). When a value of binding energy was less than zero, those proteins were considered to be spontaneously binding and interacting with each other. The lower the binding energy, the more stable the molecular conformation. The results of molecular docking were visualized using PyMOL 2.5.2 software.

### 4.14. CO-Immunoprecipitation (CO-IP) Assay

Total protein from C2C12 cells and murine tibialis tissues were extracted using RIPA buffer. We precleared cell lysate using anti-species-specific IgG beads, and incubated precleared cell lysate with PFKFB3 (Proteintech), ADK (Abcam, Cambridge, UK), MYOD1 (Proteintech), and Myosin (Sigma). Following incubation with pre-equilibrated protein A/G agarose beads, the CO-IP targets were evaluated by Western blotting.

### 4.15. Statistics

Quantitative data were presented as mean ± SEM. The statistical analysis was performed by GraphPad prism software 8.0.1. Normal distribution was determined by Kolmogorov–Smirnov test, and statistical comparisons between two groups were analyzed using two-tailed unpaired Student’s *t*-test or one- or two-way of variance (ANOVA), followed by Bonferroni’s post hoc tests when appropriate. * *p* < 0.05 was considered statistically significant.

## 5. Conclusions

In summary, this study demonstrated that adenosine dynamically distributes between extracellular and intracellular, and rapidly induces the ADK-dependent intracellular signaling pathway, which interacts with PFKFB3-mediated glycolytic metabolism to promote satellite cell activity, as well as new myofiber formation, differentiation, and fusion, and eventually promotes skeletal muscle regeneration after injury stress (Figure 8G). Adenosine triggers an ADK-dependent intracellular signaling pathway that interacts with PFKFB3-mediated glycolytic metabolism to promote newly formed myofibers development.

## Data Availability

The original contributions presented in this study are included in the article/Supplementary Materials. Further inquiries can be directed to the corresponding authors.

## Supplementary Materials

The following supporting information can be downloaded at https://www.mdpi.com/article/10.3390/ijms262412184/s1.

## Abbreviations

The following abbreviations are used in this manuscript:

AD	Adenosine
ADK	Adenosine kinase
PFKFB3	6-phosphofructo-2-kinase/fructose-2,6-biphosphatase 3
CTX	Cardiotoxin
MYOG	Myogenin
MRFs	Myogenic regulatory factors
MDGF	Macrophage-derived growth factor
TA	Tibialis anterior
FBS	Fetal bovine serum
DES	Donor equine serum
