# Supplementary material for: Adenosine Triggers an ADK-Dependent Intracellular Signaling Pathway Interacts PFKFB3-Mediated Glycolytic Metabolism to Promote Newly Formed Myofibers Development
Source: Int J Mol Sci. 2025 Dec 18;26(24):12184. doi: 10.3390/ijms262412184 (PMC12733700; doi:10.3390/ijms262412184)

**Supplementary material**

# Adenosine triggers an ADK-dependent intracellular signaling pathway interacts PFKFB3-mediated glycolytic metabolism to promote newly formed myofibers development

Xiao Wu [1†], Dawei Zeng [1†], Baojia Wang [1], Jie Liu [1], Yue Zhang [1], Cong Huang [1], Qian Nie [2], Liangqin Shi [1] and Yong Wang [1*]

[1] College of Basic Medicine, Chengdu University of Traditional Chinese Medicine, Chengdu 611137, China

[2] Hospital of Chengdu University of Traditional Chinese Medicine, School of Clinical Medicine, Chengdu University of Traditional Chinese Medicine, Chengdu 610075, China.

[†] Authors have contributed equally to this work and share first authorship.

[*] Correspondence: yongwang1008@hotmail.com (wangyong@cdutcm.edu.cn).

**Supplementary figure Legends**

**supplementary figure S1**. **(A)** Purity of Murine satellite cells (M-SC) were validated by IF staining against PAX7 antibody. **(B)** M-SCs were treated with different doses of adenosine (AD) for 6 hours (h), cell viability was determined by CCK-8 assay (*n* = 7, unpaired *t*-test). **(C)** Following adenosine treatment for 30 h, real time PCR was performed to determine transcription levels of cell cycle negatively regulated genes in M-SCs (*n* = 6; unpaired *t*-test)**. (D)** M-SC was treated with adenosine (10 μM) for 30 hours and cell cycle was evaluated by flow cytometry analysis after propidium iodide (PI) staining. Data were presented as mean ± SEM, *P < 0.05 was considered significant.

**supplementary figure S2**. **(A)** C2C12 cells were treated with adenosine for 12 hours, and real time PCR was performed to determine cell growth related genes (*n* = 6, unpaired *t*-test)**. (B)** C2C12s were treated with different doses of adenosine, cell viability was determined by CCK-8 assay (*n* = 8, unpaired *t*-test). **(C)** C2C12 cells were treated with adenosine (10 μM) for 24 hours, and the cell cycle was evaluated by flow cytometry analysis after PI staining. Data were presented as mean ± SEM, *P < 0.05 was considered significant.

**supplementary figure S3**. **(A)** Representative images of H&E staining of TA muscles on day 3 after cardiotoxin injection. **(B)** Representative images of H&E staining of TA muscles on day 5 after cardiotoxin injection.

**supplementary figure S4**. **(A)** Quantification of MYOD1 positive signals from TA muscle on day 5 after cardiotoxin injection (*n* = 6; unpaired *t*-test). **(B)** IHC staining

against MYOG antibody from TA muscle on day 5 after cardiotoxin injection. **(C)** Quantitative analysis of MYOG positive myofibers (*n* = 6; unpaired *t*-test). **(D)** Quantification of Myosin positive signals from TA muscle on day 5 after cardiotoxin injection (*n* = 6; unpaired *t*-test). **(E)** Quantitative analysis of multinucleated muscle fibers by IF staining against Myosin antibody from TA muscle on day 5 after cardiotoxin injection (*n* = 6; unpaired *t*-test). **(F)** IHC staining against PCNA antibody on slide from TA muscle on day 5 after cardiotoxin injection. Data were presented as mean ± SEM, *P < 0.05 was considered significant.

**supplementary figure S5. (A)** M-SC was treated with Adenosine (10 μM) for 48 hours, and transcription levels of adenosine receptors were determined by real time PCR (*n* = 6, unpaired *t*-test). Real time PCR performed to evaluate transcription levels of adenosine receptors and nucleoside transporters after C2C12s were treated with Adenosine for 1 hours **(B)**, 6 hours **(C)** and 12 hours **(D)** (*n* = 6, unpaired *t*-test). **(E)** Transcript levels of adenosine receptors and nucleoside transporters in TA muscle were detected by real time PCR on day 5 after cardiotoxin injection (*n* = 6; unpaired *t*-test). Data were presented as mean ± SEM, *P < 0.05 was considered significant.

**supplementary figure S6. (A)** Multiple signaling pathways were evaluated by real time PCR in M-SC after adenosine (10 μM) treatment for 24 hours (*n* = 6; unpaired *t*-test). **(B)** Quantification of ADK protein levels in M-SC after different doses of adenosine treatment for 36 hours (*n* = 3; one-way ANOVA). **(C)** M-SC were treated with adenosine (10 μM), and proteins were harvested at different time points. Western blotting was performed to determine ADK expression. Relative ADK expression was

quantified in **(D)** (*n* = 3; one-way ANOVA). **(E)** Trascript level of ADK in C2C12 was detected by real time PCR after adenosine (10 μM) treatment for 12 hours (*n* = 6; unpaired *t*-test). **(F)** Quantification of ADK protein levels in C2C12 after different doses of adenosine treatment for 24 h (*n* = 3; one-way ANOVA). **(G)** C2C12 were treated with adenosine (10 μM), proteins were harvested different time points, and Western blotting was performed to determine the expression of ADK. ADK protein was quantified in **(H)** (*n* = 3; one-way ANOVA). **(I)** IHC staining against ADK antibody on TA muscle on day 5 after cardiotoxin injection. **(J)** The relative expression of ADK was quantified in **(I)** (*n* = 6; unpaired *t*-test). Quantitative data were presented as mean ± SEM, *P < 0.05 was considered significant.

**supplementary figure S7. (A)** Quantification of PFKFB3 protein levels after adenosine treatment for different time points (*n* = 3; one-way ANOVA). **(B)** M-SC were treated with different doses of adenosine for 36 hours, and expression levels of PFKFB3 was evaluated by Western blotting, and relative protein level of PFKFB3 was quantified in **(C)** (*n* = 3; one-way ANOVA). **(D)** C2C12 were treated with different doses of adenosine for 24 hours, and the expression levels of PFKFB3 was evaluated by Western blotting, and the relative protein level of PFKFB3 was quantified in **(E)** (*n* = 3; one-way ANOVA). **(F)** Quantification of PFKFB3 protein levels after adenosine treatment in C2C12 for different time points (*n* = 3; one-way ANOVA). **(G)** Quantification of PFKFB3 expression within newly formed myofibers based on integrated optical density (IOD) on day 5 after cardiotoxin injection (*n* = 6; unpaired *t*-test). **(H)** IF staining against PFKFB3 and Myosin antibodies on TA muscle

on day 5 after cardiotoxin injection, newly formed myofibers were visualized by staining with Myosin antibody. Data were presented as mean ± SEM, *P < 0.05 was considered significant.

**supplementary figure S8. (A)** Binding affinity between ADK and PFKFB3 evaluated by Molecular docking. **(B)** Total protein was extracted from C2C12 and CO-IP performed to determine interaction between ADK and PFKFB3. **(C)** Nucleotide sequence used to silence ADK or PFKFB3 by siRNAs targeting murine ADK or PFKFB3. **(D)** C2C12 were transfected with Scrambled siRNA and siRNAs targeting murine ADK (S1, S2, S3). Silence efficiency of ADK was validated by real time PCR (*n* = 6, unpaired *t*-test, S2 selected for further study). **(E)** C2C12 were transfected with Scrambled siRNA and siRNAs targeting murine ADK, and real time PCR was performed to detect skeletal muscle differentiation associated genes (*n* = 6; unpaired *t*-test). **(F)** C2C12 were transfected with Scrambled siRNA and siRNAs targeting murine PFKFB3 (S1, S2, S3), and real time PCR was performed to validate silence efficiency of PFKFB3 (*n* = 6, unpaired *t*-test, S2 selected for further study). **(G)** C2C12 were transfected with small interfering RNA targeting PFKFB3, differentiation was induced by DES for 2 days, and real time PCR was performed to detect skeletal muscle differentiation associated genes (*n* = 6; unpaired *t*-test). Data presented as mean ± SEM, *P < 0.05 was considered significant.

**supplementary figure S9. (A)** C2C12 cells were treated with 3PO (20 μM), an inhibitor of PFKFB3, differentiation was induced by DES for 30 hours, and expressions of MYOD1was determined by Western blot. The relative expression level

of MYOD1 was quantified in (**B**) (*n* = 3, one-way ANOVA). **(C)** After treated with 3PO (20 μM), following adenosine (10 μM) treatment for 12 hours, and following incubated with BrdU labeling reagent for 6 hours, IF staining was performed to determine BrdU incorporation. Quantification of BrdU positive cells (*n* = 6; unpaired *t*-test). **(D)** C2C12s were treated by 3PO, following adenosine treatment. Quantification of migrated C2C12 cells (*n* = 6; unpaired *t*-test). **(E)** After transfection with siRNA, C2C12 differentiation was induced by DES contained 3PO (20 μM) and adenosine (10 μM) for 3 days, crystal violet staining performed to visualize fusion of myofibers (arrows: fusion of myofibers). **(F)** C2C12 were transfected with Scrambled siRNA and siRNAs targeting murine ADK, real time PCR performed to determine PFKFB3 transcription level (*n* = 6; unpaired *t*-test). **(G)** C2C12 were transfected with Scrambled siRNA and siRNAs targeting murine PFKFB3, real time PCR performed to determine ADK transcription level (*n* = 6; unpaired *t*-test). Data presented as mean ± SEM, *P < 0.05 was considered significant.

**supplementary figure S10.** IF staining against MAC2 antibody was performed to determine Macrophages infiltration on day 3 **(A)**, day 5 **(B)** and day 7 **(C)** after cardiotoxin injection. Real time PCR was performed on day 3 **(D)**, day 5 **(E)** after cardiotoxin injection to determine transcription levels of inflammatory regulating genes (*n* = 6, unpaired *t*-test). Quantitative data presented as mean ± SEM, *P < 0.05 was considered significant.

Supplementary figure S1

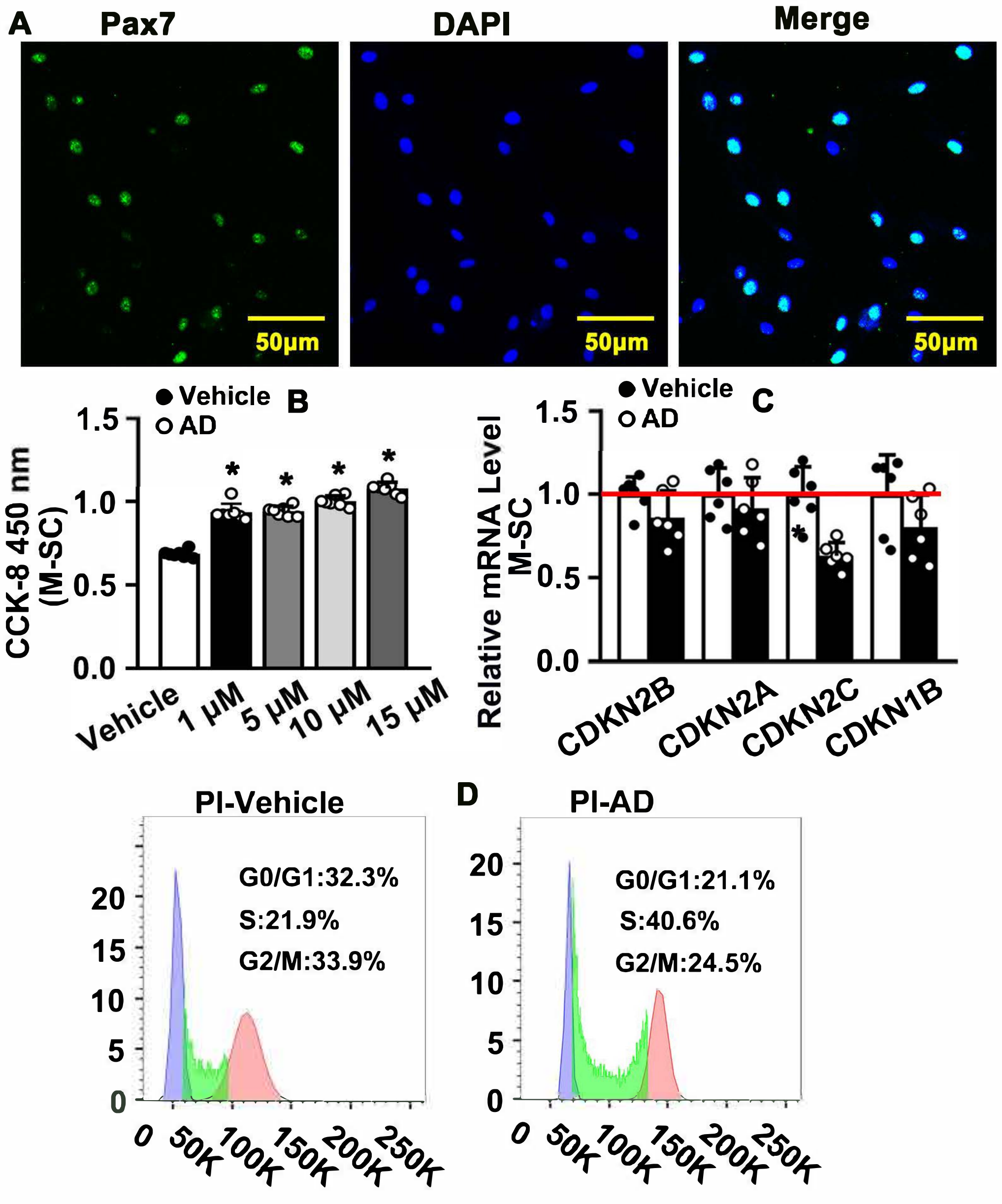

Supplementary figure S2

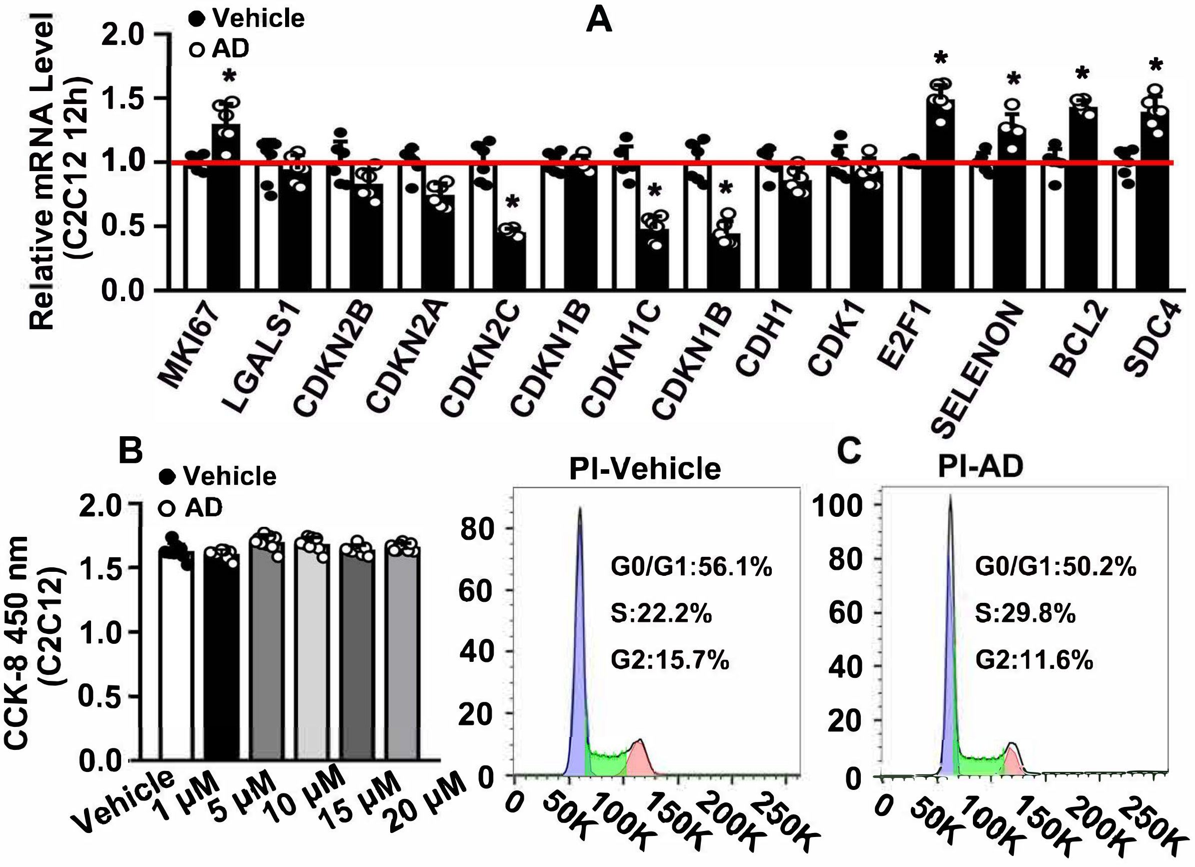

## Supplementary figure S3

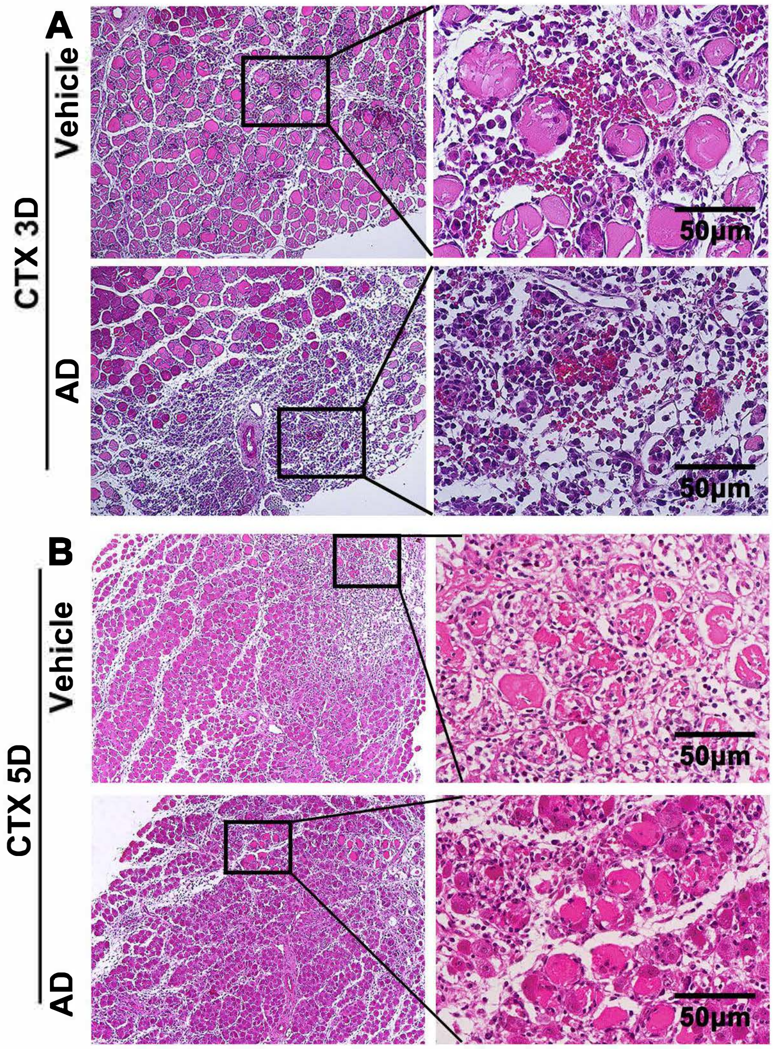

Supplementary figure S4

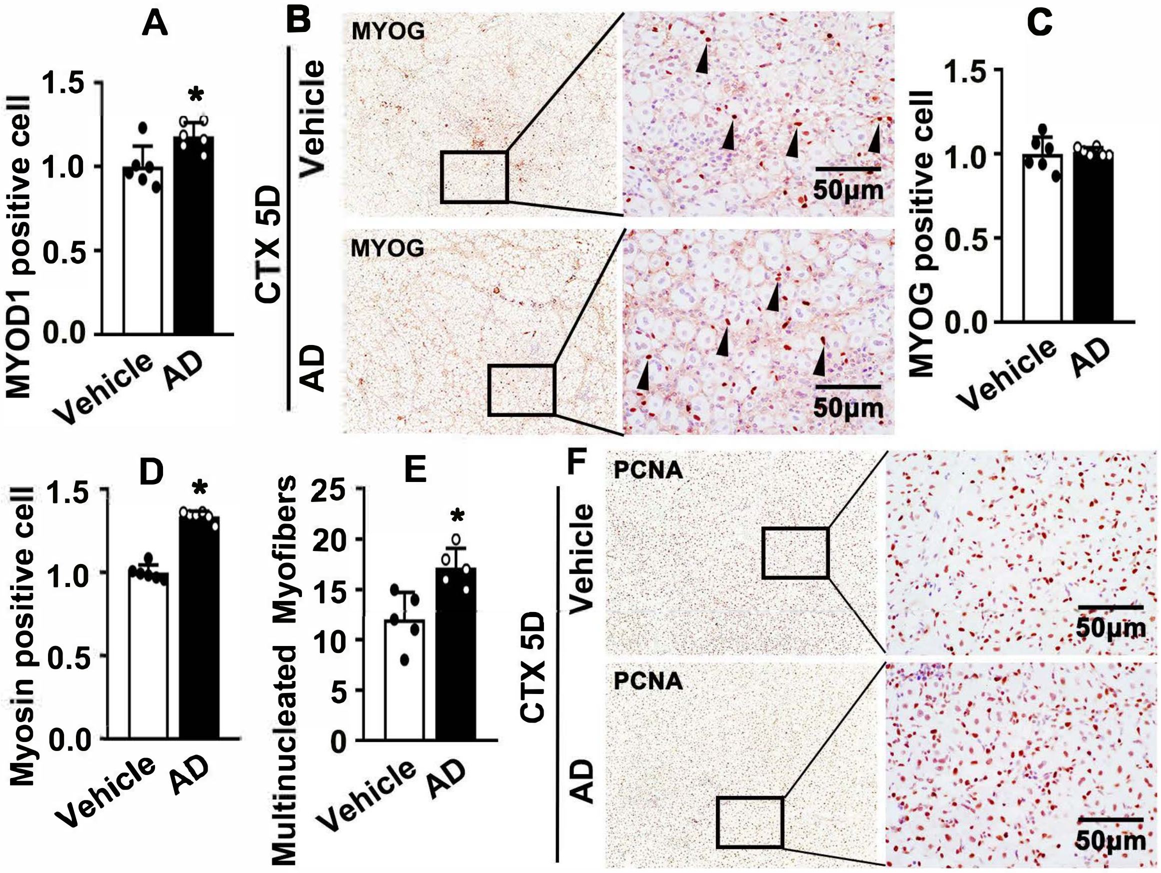

**Supplementary figure S5**

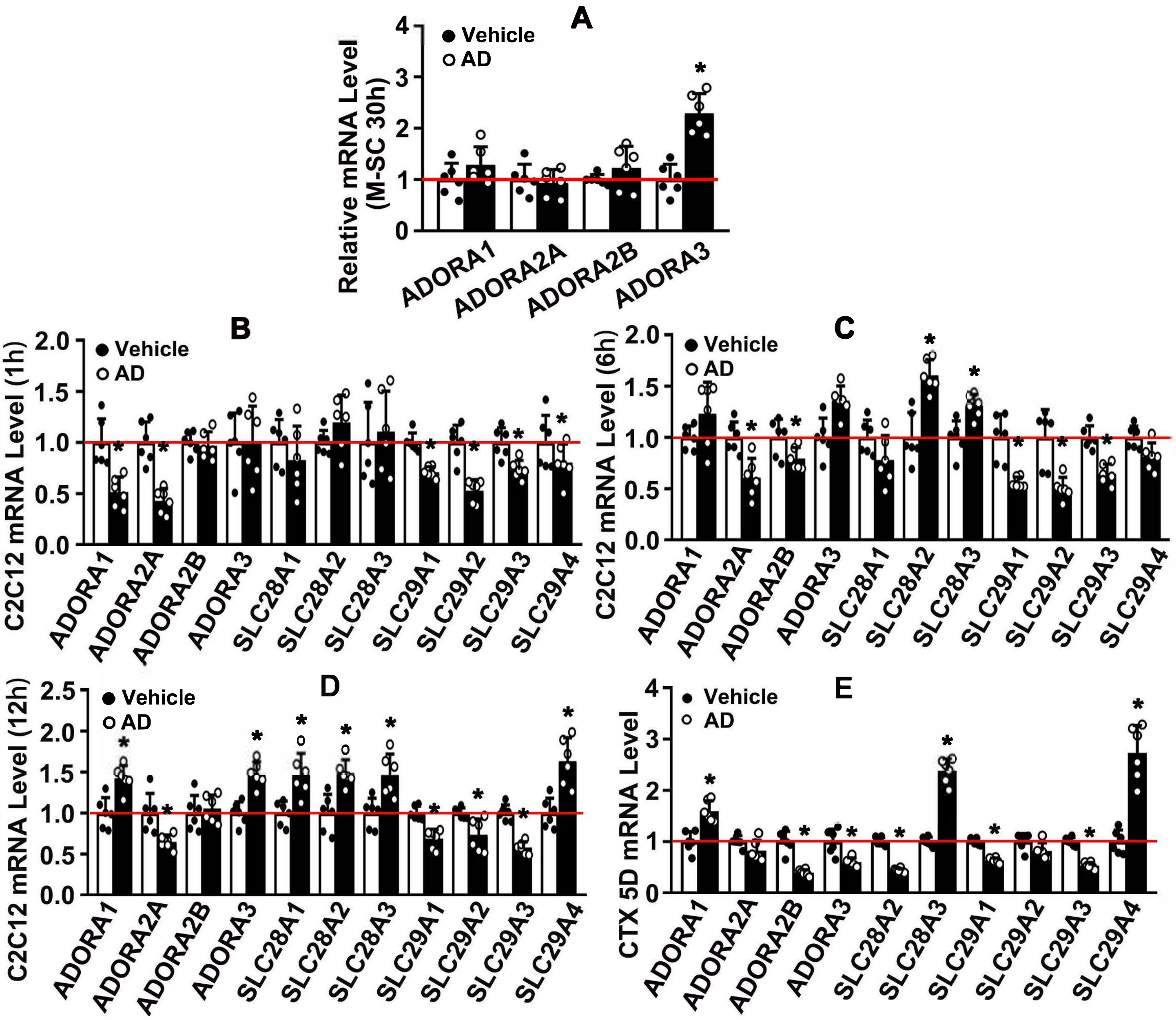

Supplementary figure S6

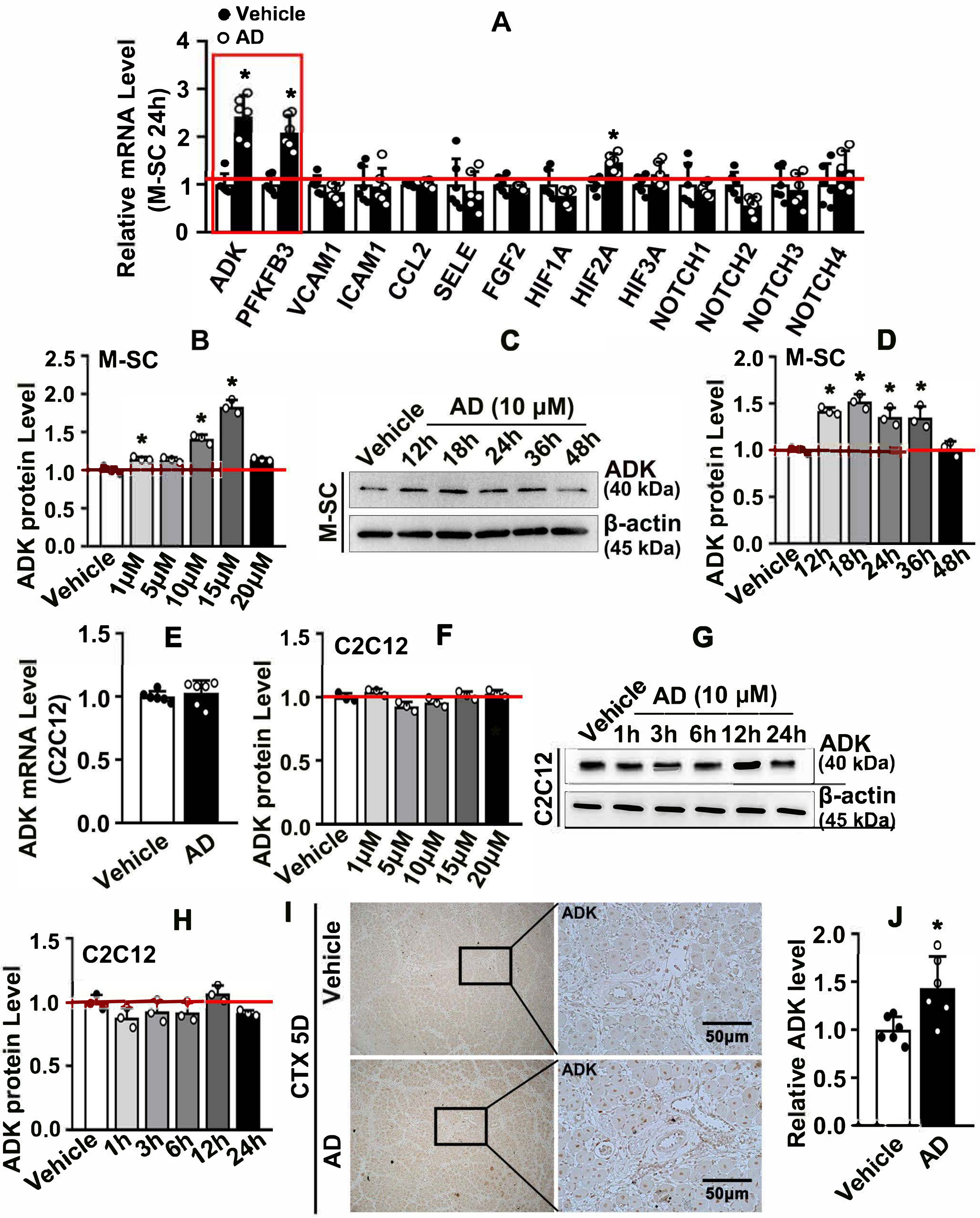

Supplementary figure S7

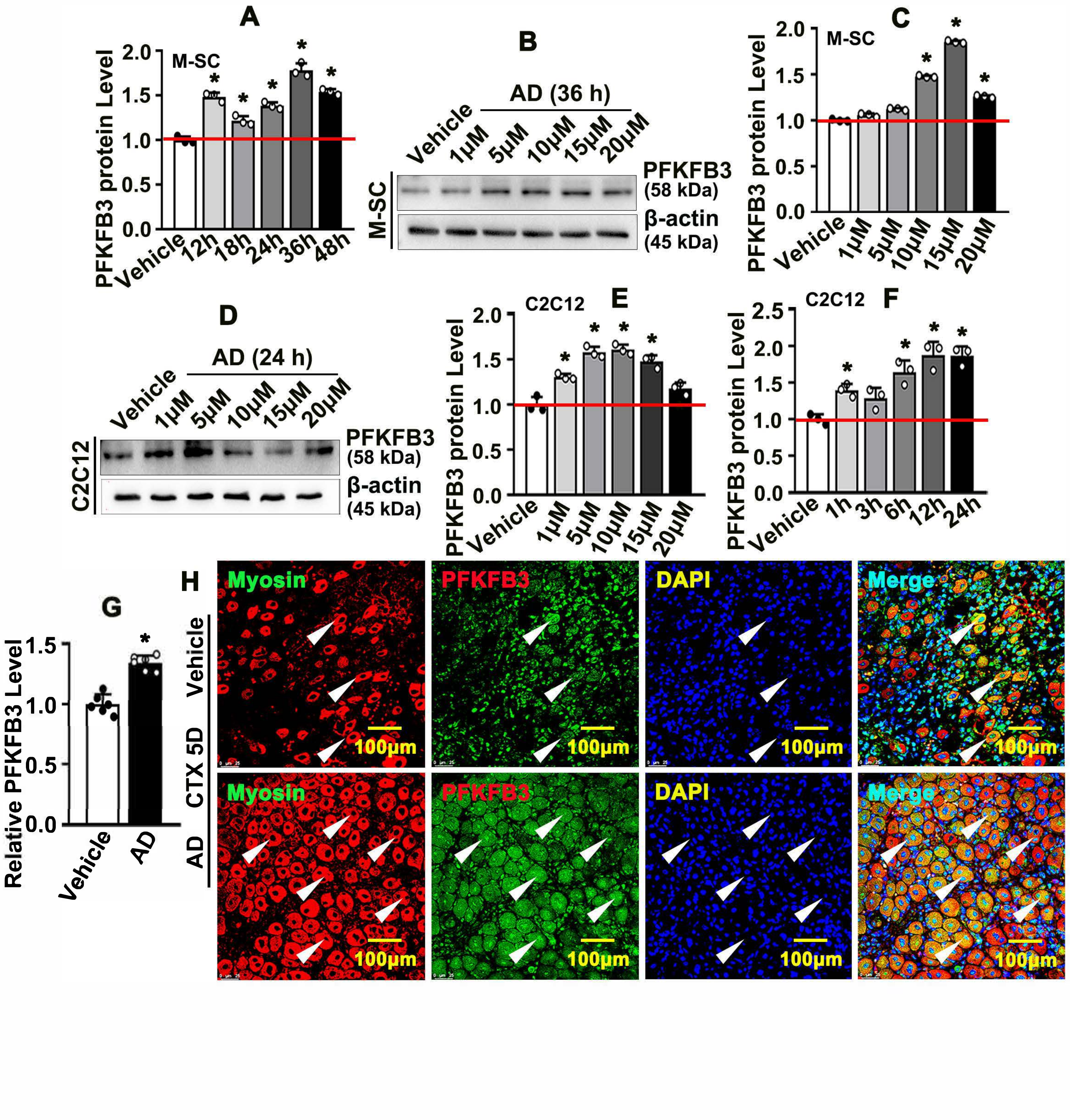

## Supplementary figure S8

### A

| Molecule name | Target | PDB ID | Binding affinity (KJ·mol-1) |
|---|---|---|---|
| Adenosine | *ADK* | 1bx4 | -7.2 |
| | *PFKFB3* | 6hvi | -6.6 |

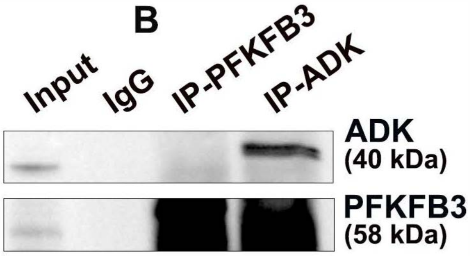


### C

| *Gene Name* | *Species* | *Sequence* |
|---|---|---|
| si-ADK | Mouse | F: 5，-GCCGCCAAUUGUUACAAGATT-3， |
| | Mouse | R: 5，-UCUUGUAACAAUUGGCGGCTT-3， |
| si-PFKFB3 | Mouse | F: 5，-GAGCCUGUGAUCAUGGAAUTT-3， |
| | Mouse | R: 5，-AUUCCAUGAUCACAGGCUCTT-3， |

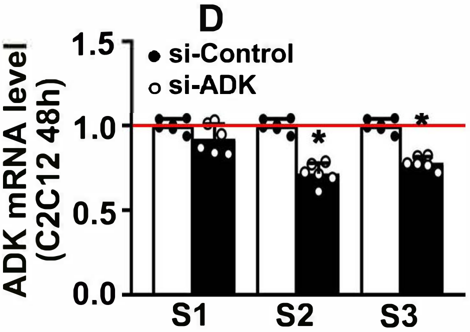


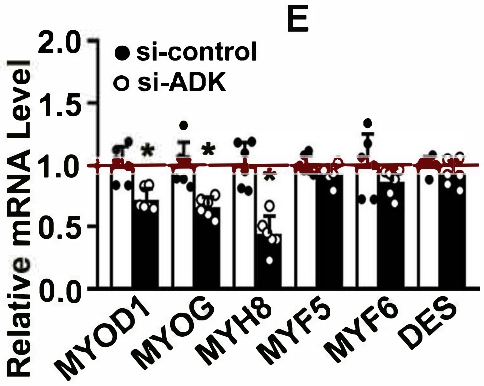


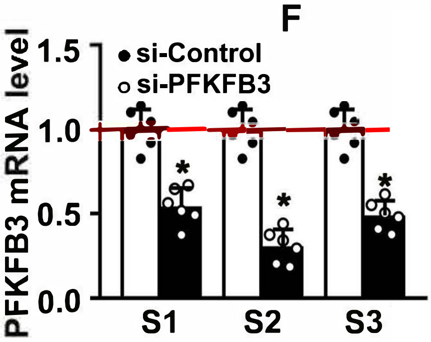


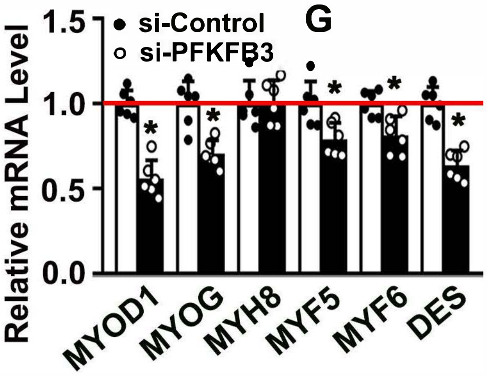

Supplementary figure S9

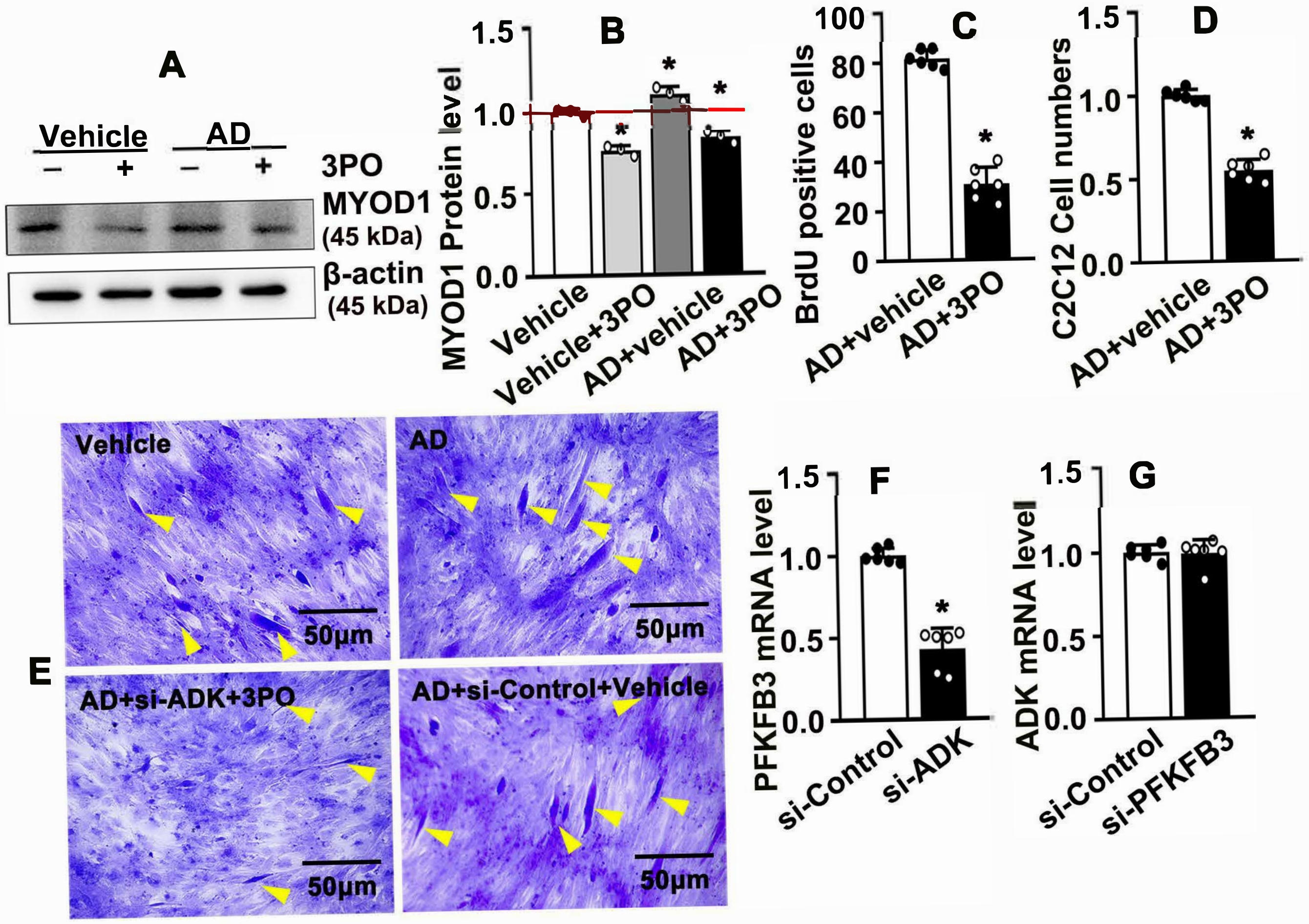

Supplementary figure S10

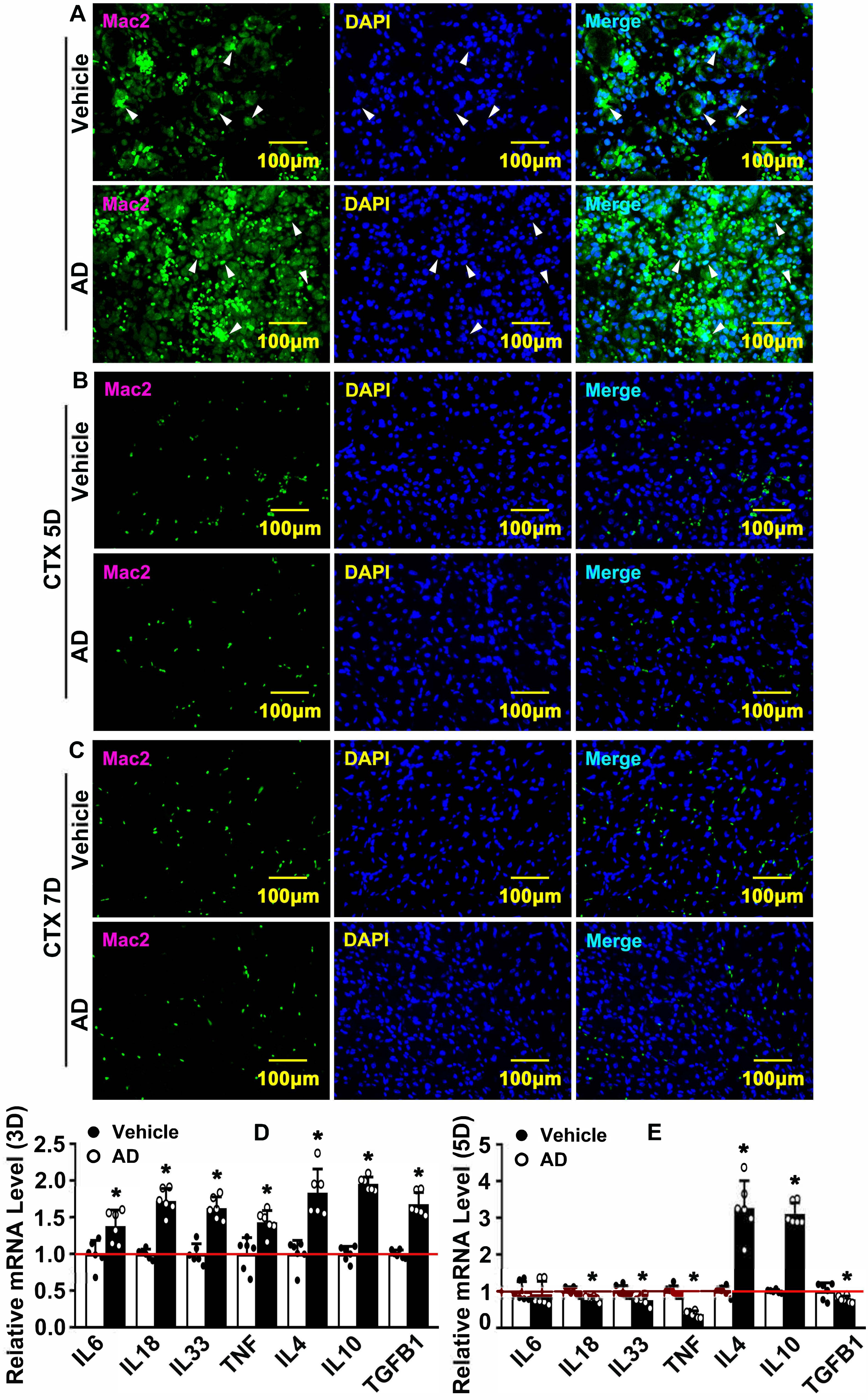

Supplement: Supplementary file 1 [file ijms-26-12184-s001.zip › ijms-4005734-supplementary.pdf]
